# Supplementary material for: PRMT5-mediated arginine methylation activates AKT kinase to govern tumorigenesis
Source: Nat Commun. 2021 Jun 8;12:3444. doi: 10.1038/s41467-021-23833-2 (PMC8187744; doi:10.1038/s41467-021-23833-2)
Supplement: Supplementary file 1 — Supplementary Information [file 41467_2021_23833_MOESM1_ESM.pdf]

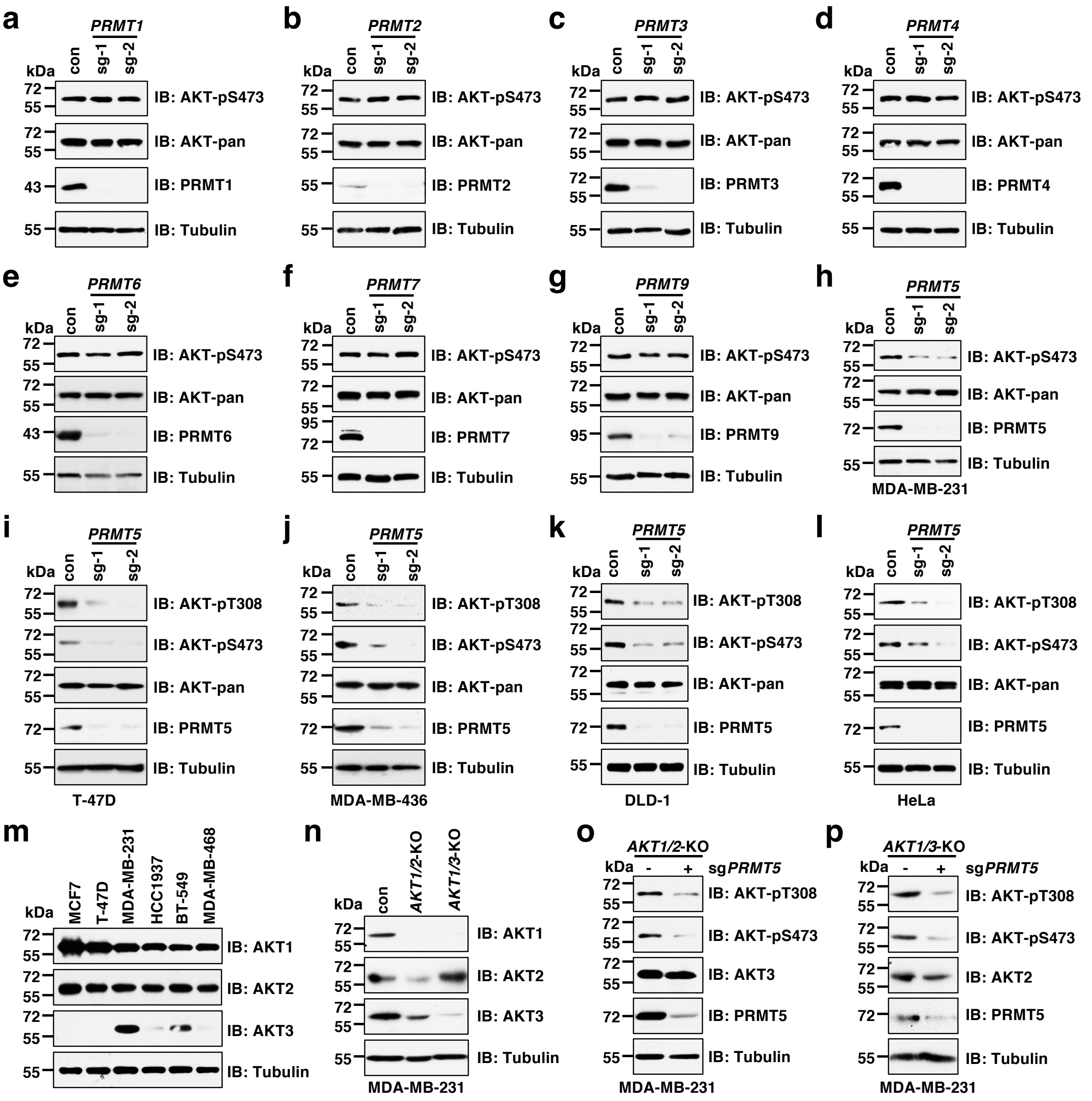
a
PRMT1
con
sg-1
sg-2
kDa
IB: AKT-pS473
IB: AKT-pan
IB: PRMT1
IB: Tubulin
b
PRMT2
IB: PRMT2
c
PRMT3
IB: PRMT3
d
PRMT4
IB: PRMT4
e
PRMT6
IB: PRMT6
f
PRMT7
IB: PRMT7
g
PRMT9
IB: PRMT9
h
PRMT5
IB: PRMT5
MDA-MB-231
i
IB: AKT-pT308
T-47D
j
MDA-MB-436
k
DLD-1
l
HeLa
m
MCF7
T-47D
MDA-MB-231
HCC1937
BT-549
MDA-MB-468
IB: AKT1
IB: AKT2
IB: AKT3
n
AKT1/2-KO
AKT1/3-KO
o
sgPRMT5
p

Supplementary Fig. 1 PRMT5, but not other PRMTs, controls AKT activation in cancer cells. **a-g** Immunoblot (IB) analysis of whole cell lysates (WCL) derived from MCF7 cells infected with *PRMTs*-targeting lentiCRISPR virus (sg-1 and sg-2) and GFP-targeting lentiCRISPR virus as a negative control (con). **h-l** IB analysis of WCL derived from MDA-MB-231, T-47D, MDA-MB-436, DLD-1 and HeLa cells infected with *PRMT5*-targeting lentiCRISPR virus. **m** IB analysis of WCL derived from various breast cancer cells. **n** IB analysis of WCL derived from MDA-MB-231 cells depleted *AKT1/2* (*AKT1/2*-KO) or *AKT1/3* (*AKT1/3*-KO). Cells were infected with *AKT1/2 or AKT1/3*-targeting lentiCRISPR virus. **o, p** IB analysis of WCL derived from *AKT1/2*-KO or *AKT1/3*-KO MDA-MB-231 cells depleted *PRMT5*. Cells in **a-l** and **n-p** were selected with 2 μg/ml puromycin for 4 days to eliminate the non-infected cells before harvesting. Similar results were obtained in $n \geq 2$ independent experiments in **a-p**. Uncropped immunoblots are provided in Source Data file.

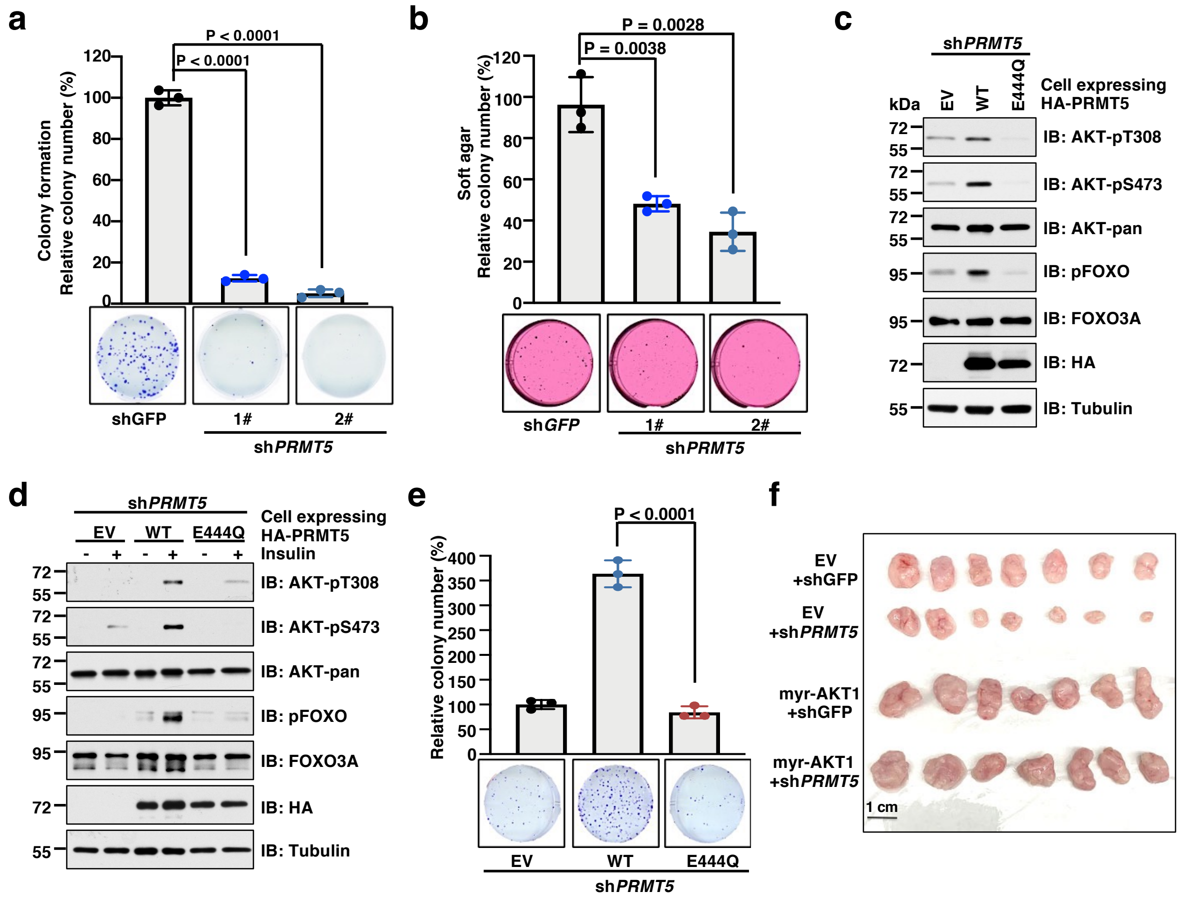

Supplementary Fig. 2 PRMT5 promotes colony formation and AKT activation in an enzymatic activity-dependent manner. a, **b** *PRMT5*-depleted MCF7 cells were subjected to colony formation (**a**) and soft agar assays (**b**). Representative images are shown (bottom), and relative colony numbers are plotted (top). Data are shown as the mean ± s.d. of n = 3 independent experiments. Statistical significance was determined by two-tailed Student's t-test. **c, d** IB analysis of WCL derived from *PRMT5*-depleted MCF7 cells expressing PRMT5-WT or E444Q. Where indicated, cells were serum-starved for 16 hr and then treated with 100 nM insulin for 0 or 30 min before harvesting. **e** *PRMT5*-depleted MCF7 cells were re-introduced PRMT5-WT or E444Q mutant and subjected to colony formation assays. Representative images are shown (bottom), and relative colony numbers are plotted (top). Data are shown as the mean ± s.d. of n =3 independent experiments. Statistical significance was determined by two-tailed Student's t-test. **f** Tumors derived from xenograft assays. Similar results were obtained in n = 2 independent experiments in **c, d**. Uncropped immunoblots and statistical source data are provided in Source Data files.

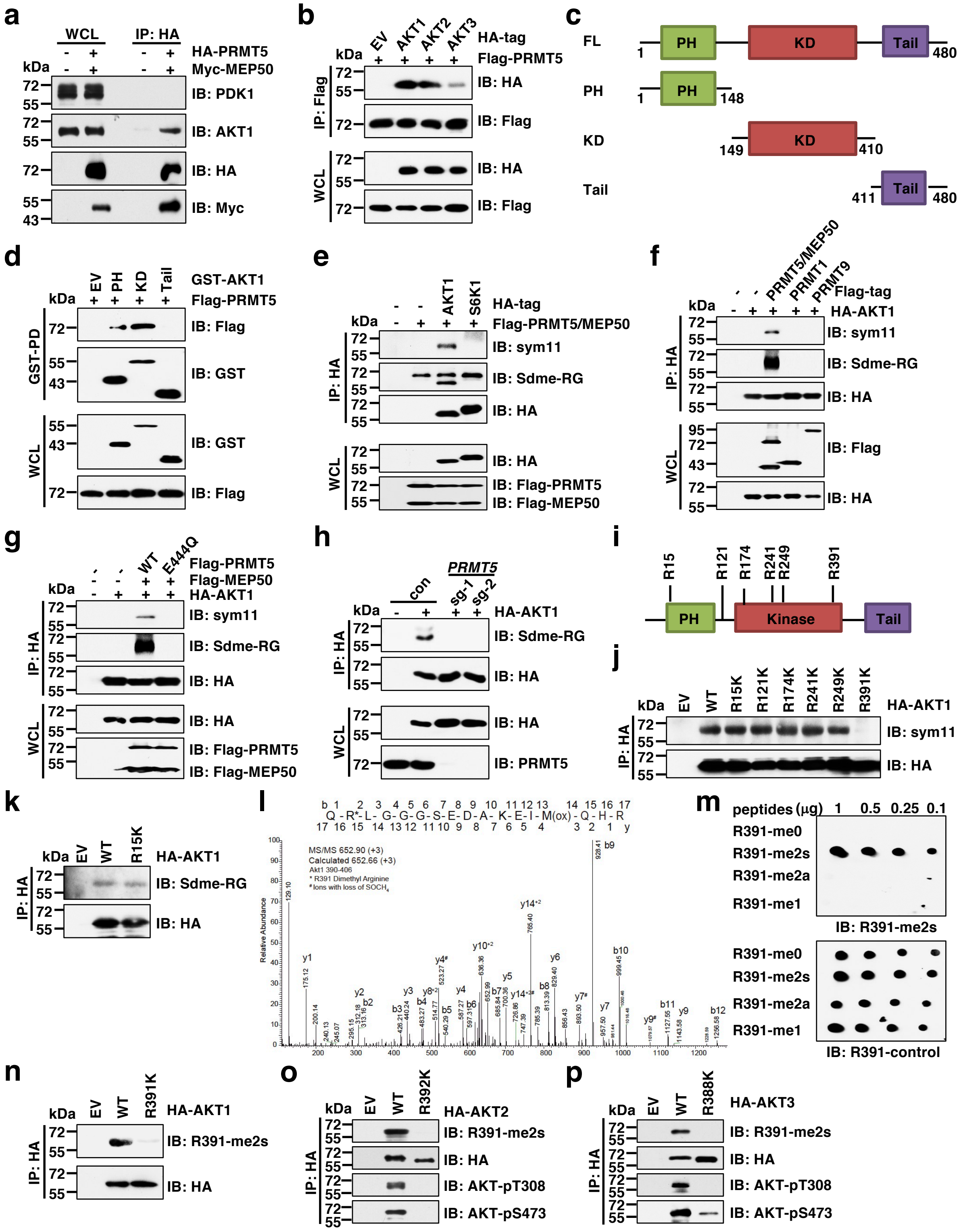

a
WCL
IP: HA
HA-PRMT5
Myc-MEP50
kDa
IB: PDK1
IB: AKT1
IB: HA
IB: Myc
b
EV
AKT1
AKT2
AKT3
HA-tag
Flag-PRMT5
IP: Flag
WCL
IB: HA
IB: Flag
c
FL
PH
KD
Tail
1
148
149
410
411
480
d
EV
PH
KD
Tail
GST-AKT1
Flag-PRMT5
GST-PD
WCL
IB: Flag
IB: GST
e
AKT1
S6K1
HA-tag
Flag-PRMT5/MEP50
IP: HA
WCL
IB: sym11
IB: Sdme-RG
IB: HA
IB: Flag-PRMT5
IB: Flag-MEP50
f
PRMT5/MEP50
PRMT1
PRMT9
Flag-tag
HA-AKT1
IB: sym11
IB: Sdme-RG
IB: HA
IB: Flag
g
WT
E444Q
Flag-PRMT5
Flag-MEP50
HA-AKT1
IB: sym11
IB: Sdme-RG
IB: HA
IB: Flag-PRMT5
IB: Flag-MEP50
h
con
PRMT5
sg-1
sg-2
HA-AKT1
IB: Sdme-RG
IB: HA
IB: PRMT5
i
R15
R121
R174
R241
R249
R391
PH
Kinase
Tail
j
EV
WT
R15K
R121K
R174K
R241K
R249K
R391K
HA-AKT1
IB: sym11
IB: HA
k
EV
WT
R15K
HA-AKT1
IB: Sdme-RG
IB: HA
l
Q - R*- L- G- G- G- S- E- D- A- K- E- I - M(ox) - Q - H - R
MS/MS 652.90 (+3)
Calculated 652.66 (+3)
Akt1 390-406
* R391 Dimethyl Arginine
Relative Abundance
m
peptides (μg) 1 0.5 0.25 0.1
R391-me0
R391-me2s
R391-me2a
R391-me1
IB: R391-me2s
IB: R391-control
n
EV
WT
R391K
HA-AKT1
IB: R391-me2s
IB: HA
o
R392K
HA-AKT2
IB: R391-me2s
IB: HA
IB: AKT-pT308
IB: AKT-pS473
p
R388K
HA-AKT3
IB: R391-me2s
IB: HA
IB: AKT-pT308
IB: AKT-pS473

Supplementary Fig. 3 PRMT5 interacts with and methylates AKT in cells. a, b IB analysis of WCL and immunoprecipitation (IP) products derived from HEK293T cells transfected with indicated constructs. **c** Schematic presentation of the AKT1 domains. **d** IB analysis of WCL and GST-pulldown products derived from HEK293 cells transfected with indicated constructs. **e-h** IB analysis of WCL and IP products derived from HEK293 cells transfected with indicated constructs. **i** Schematic presentation of the putative methylated residues of AKT1. **j** IB analysis of IP products derived from HEK293 cells transfected with indicated constructs. **k** IB analysis of IP products derived from MCF7 cells expressing AKT1-WT or AKT1-R15K. **l** Mass spectrometry analysis of AKT1-R391 dimethylation. **m** Dot blot assays were performed to examine the specificity of AKT1-R391-me2s antibody. **n-p** IB analysis of IP products derived from HEK293T cells transfected with indicated constructs. Similar results were obtained in $n \geq 2$ independent experiments in **a, b, d-h, j, k** and **m-p**. Uncropped immunoblots are provided in Source Data file.

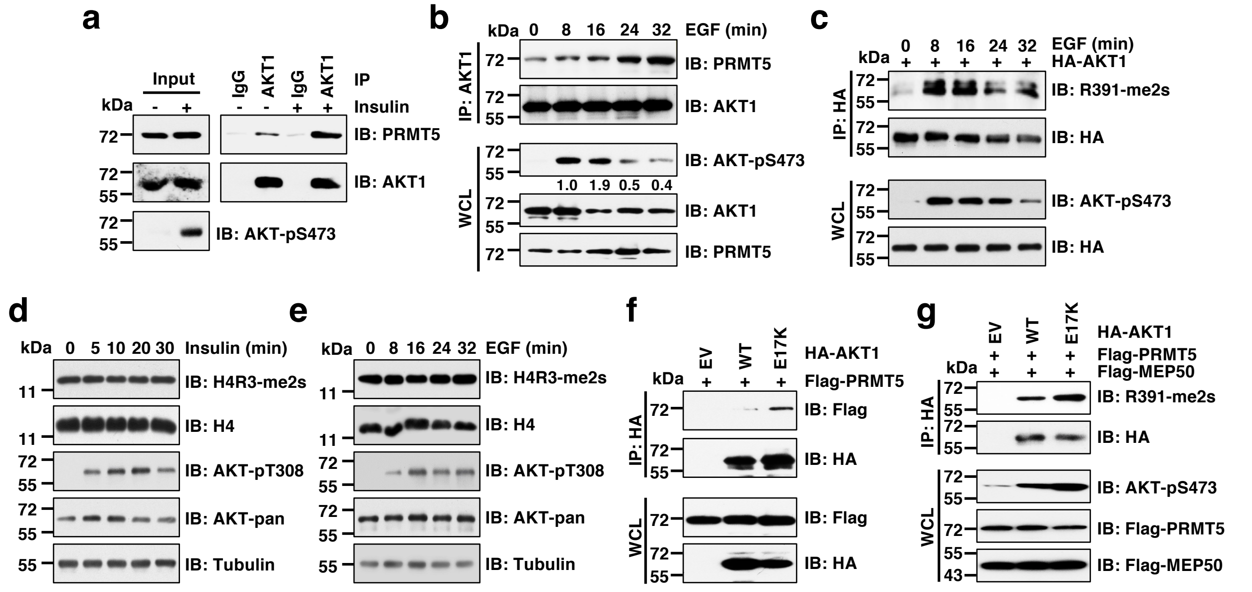

Supplementary Fig. 4 Growth factors promote AKT methylation through enhancing its interaction with PRMT5. a, b IB analysis of WCL and IP products derived from MCF7 cells. Cells were serum-starved for 16 hr and treated with 100 nM insulin for 60 min or 100 ng/mL EGF for indicated time periods before harvesting. The number represents the ratio of AKT-pS473/AKT1, which is normalized to the timepoint 8 min. **c** IB analysis of WCL and IP products derived from MCF7 cells transfected with HA-AKT1. The cells were serum-starved for 16 hr and then treated with 100 ng/mL EGF for indicated time period before harvesting. **d, e** IB analysis of WCL and histone derived from MCF7 cells. Cells were serum-starved for 16 hr and treated with 100 nM insulin or 100 ng/mL EGF for indicated time periods before harvesting. **f, g** IB analysis of WCL and IP products derived from HEK293T cells transfected with indicated constructs. Similar results were obtained in n ≥ 2 independent experiments in **a-g**. Uncropped immunoblots are provided in Source Data file.

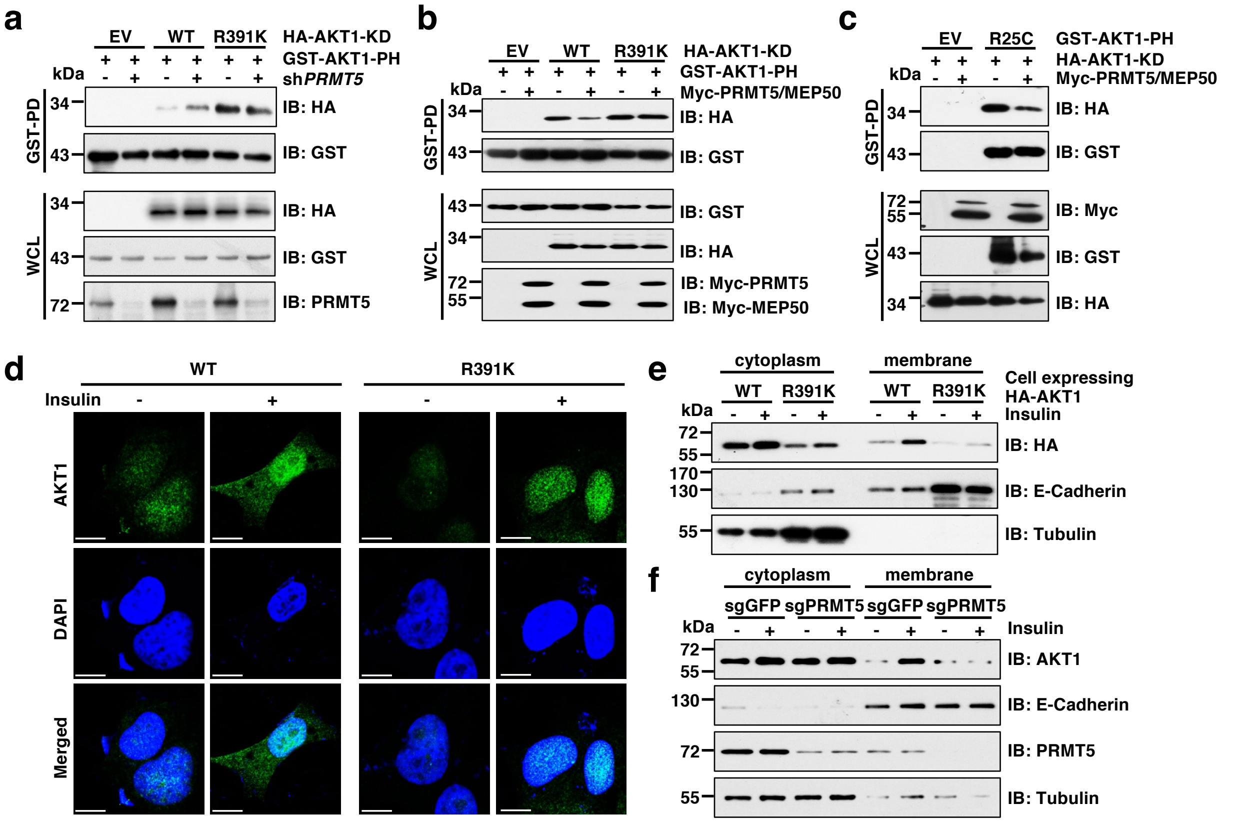

Supplementary Fig. 5 AKT1-R391 methylation releases PH-in confirmation and promotes AKT1 membrane translocation. a IB analysis of WCL and GST-pulldown products derived from *PRMT5*-depleted HEK293T cells transfected with HA-AKT1-KD and GST-AKT1-PH constructs. **b, c** IB analysis of WCL and GST-pulldown products derived from HEK293T cells transfected with indicated constructs. **d** Immunofluorescence analysis of AKT1 in DLD-1-*AKT1/2*$^{-/-}$ cells expressing AKT1-WT or AKT1-R391K. Cells were serum-starved for 16 hr and then treated with 100 nM insulin for 10 min. Scale bar, 10 µm. Similar results were obtained in n = 2 independent experiments. **e, f** IB analysis of cell fractionations derived from DLD-*AKT1/2*$^{-/-}$ cells expressing AKT1-WT or AKT1-R391K (**e**) and MCF7 depleted *PRMT5* (**f**). Similar results were obtained in n ≥ 2 independent experiments in **a-c, e** and **f**. Uncropped immunoblots are provided in Source Data file.

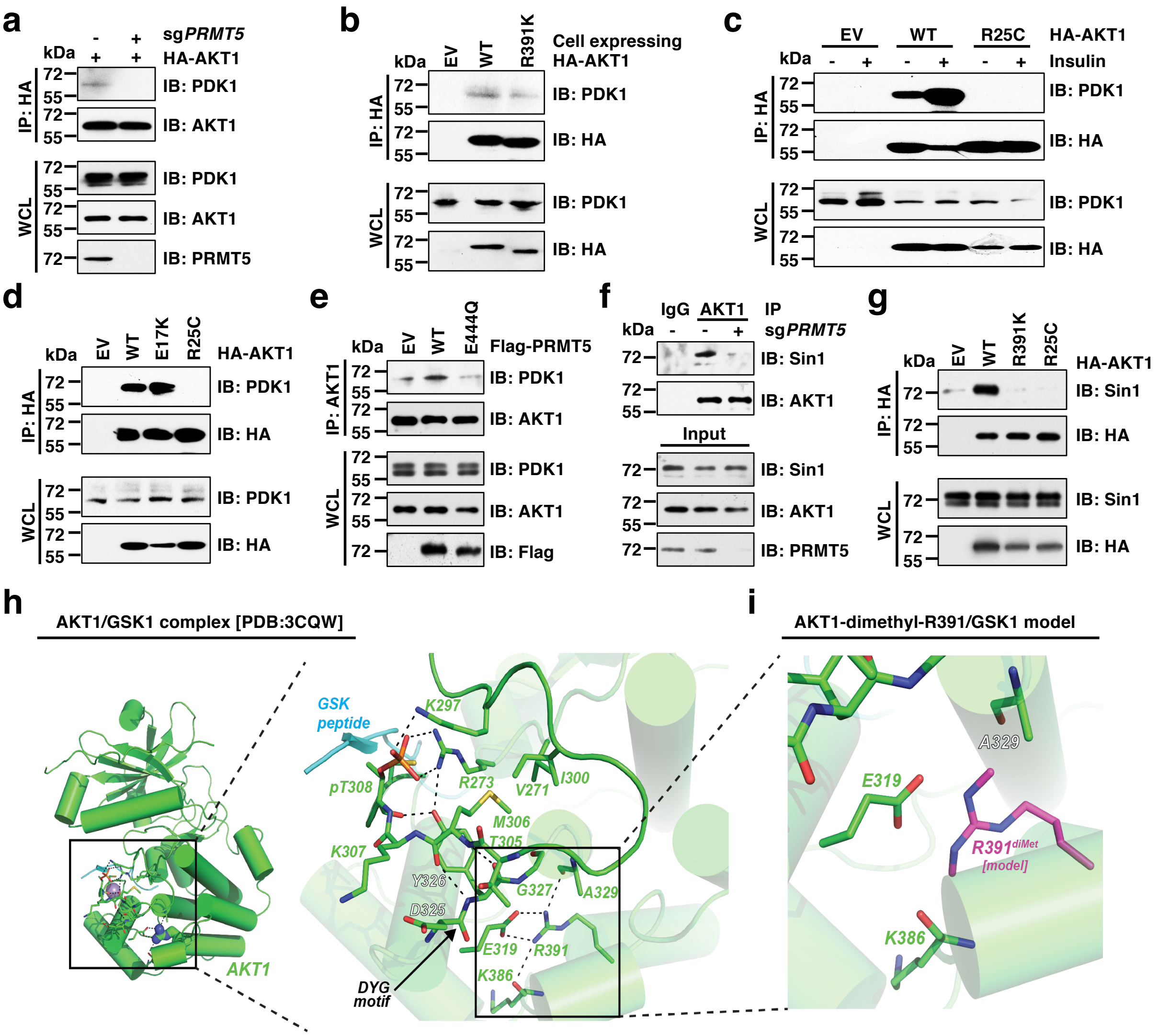

a
sgPRMT5
HA-AKT1
kDa
IP: HA
WCL
IB: PDK1
IB: AKT1
IB: PRMT5
b
Cell expressing HA-AKT1
EV
WT
R391K
IB: HA
c
EV
WT
R25C
HA-AKT1
Insulin
d
E17K
R25C
e
Flag-PRMT5
E444Q
IP: AKT1
IB: Flag
f
IgG
AKT1
IP
Input
IB: Sin1
g
h
AKT1/GSK1 complex [PDB:3CQW]
GSK peptide
K297
pT308
R273
V271
I300
M306
T305
K307
Y326
D325
G327
A329
E319
R391
K386
DYG motif
AKT1
i
AKT1-dimethyl-R391/GSK1 model
R391diMet [model]

Supplementary Fig. 6 Deficiency in AKT1-R391 methylation suppresses AKT interaction with PDK1 and mTORC2. a-d IB analysis of WCL and IP products derived from *PRMT5*-depleted MCF7 cells transfected with HA-AKT1 (**a**) or derived from MCF7 cells stably expressing AKT1-WT or R391K mutant (**b**) or derived from HEK293T cells transfected with indicated constructs (**c, d**). **e-g** IB analysis of WCL and IP products derived from MCF7 cells stably expressing PRMT5 (**e**) or derived from *PRMT5*-depelted MCF7 cells (**f**) or derived from HEK293T cells transfected with indicated constructs (**g**). **h** The structure of AKT1 in complex with a GSK1 substrate peptide and small molecule inhibitor (PDB: 3CQW) is shown as cartoon representation. An overview of the structure is shown in the left panel and a magnified view of the region around R391 of AKT1 is shown in the right panel. Amino acids involved in the network of interactions between R391 and the connection to the DYG motif and activation loop of AKT1, including pT308, are shown as sticks. Carbon atoms (green), nitrogen (blue), oxygen (blue) and sulfur (yellow). The GSK peptide is colored cyan. **i** Symmetrically dimethylated R391 was modeled onto the aforementioned structure. The model shows that the network of interactions that R391 participates in is disrupted upon methylation due to loss of hydrogen bond donors and steric clashes. Similar results were obtained in n ≥ 2 independent experiments in **a-g**. Uncropped immunoblots are provided in Source Data file.

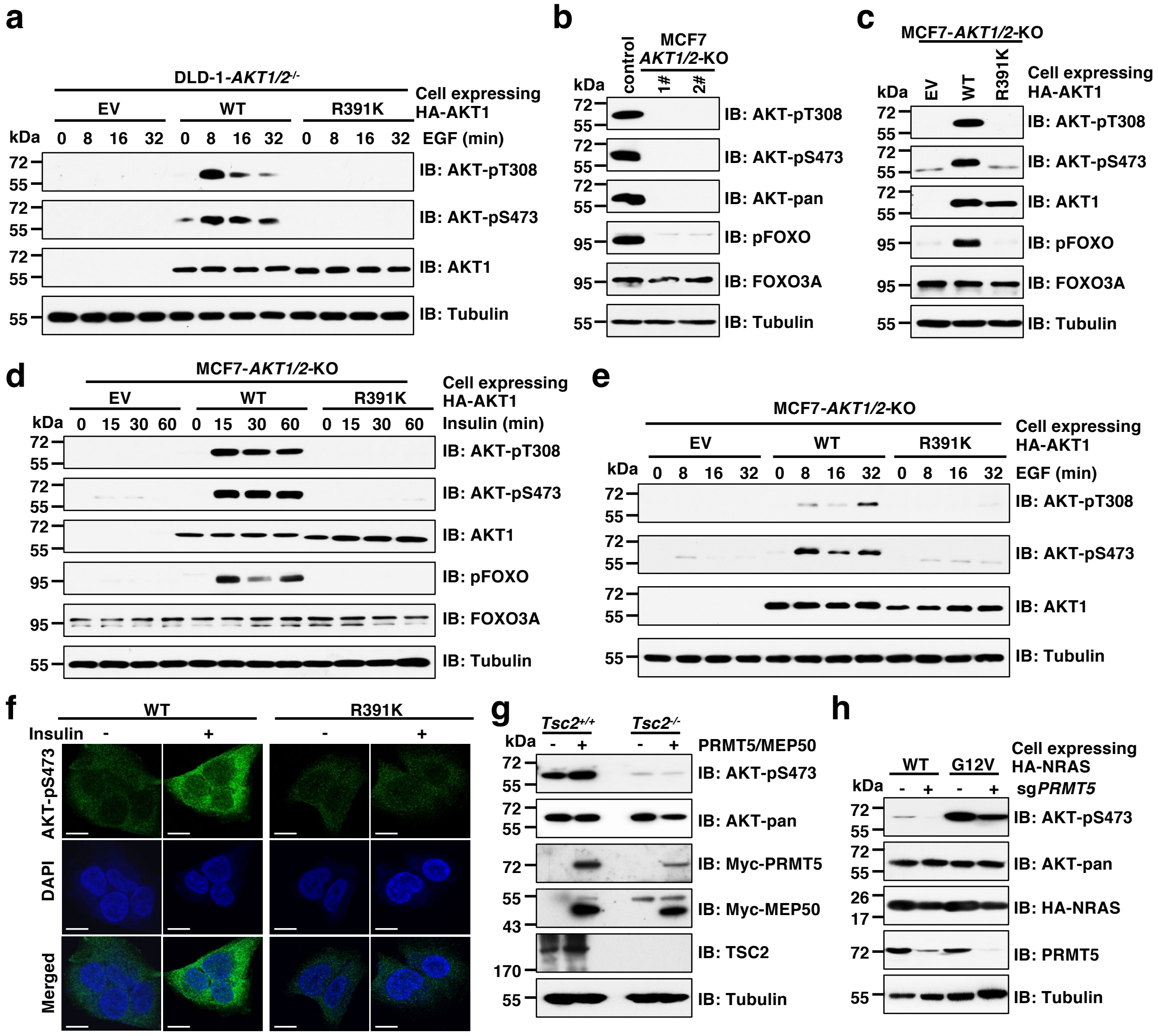

Supplementary Fig. 7 Inhibition of AKT1-R391 methylation suppresses AKT activation. a IB analysis of WCL derived from DLD-1-*AKT1/2*$^{-/-}$ cells reconstituted with AKT1-WT or AKT1-R391K mutant. The cells were serum-starved for 16 hr and then treated with 100 ng/mL EGF before harvesting. **b** IB analysis of WCL derived from MCF7-*AKT1/2*-KO cells. **c-e** IB analysis of WCL derived from MCF7-*AKT1/2*-KO cells reconstituted with AKT1-WT or AKT1-R391K mutant. Where indicated, cells were serum-starved for 16 hr and then treated with 100 nM insulin or 100 ng/mL EGF for indicated time period before harvesting. **f** Immunofluorescence analysis of AKT-pS473 in DLD-1-*AKT1/2*$^{-/-}$ cells reconstituted with AKT1-WT or AKT1-R391K mutant. Cells were serum-starved for 16 hr and treated with 100 nM insulin for 10 min. Scale bar, 10 μm. Similar results were obtained in n = 2 independent experiments. **g, h** IB analysis of WCL derived from *Tsc2*$^{+/+}$ or *Tsc2*$^{-/-}$ MEFs or MCF7 cells stably expressing NRAS-WT or NRAS-G12V mutant. Similar results were obtained in n ≥ 2 independent experiments in **a-e, g** and **h**. Uncropped immunoblots are provided in Source Data file.

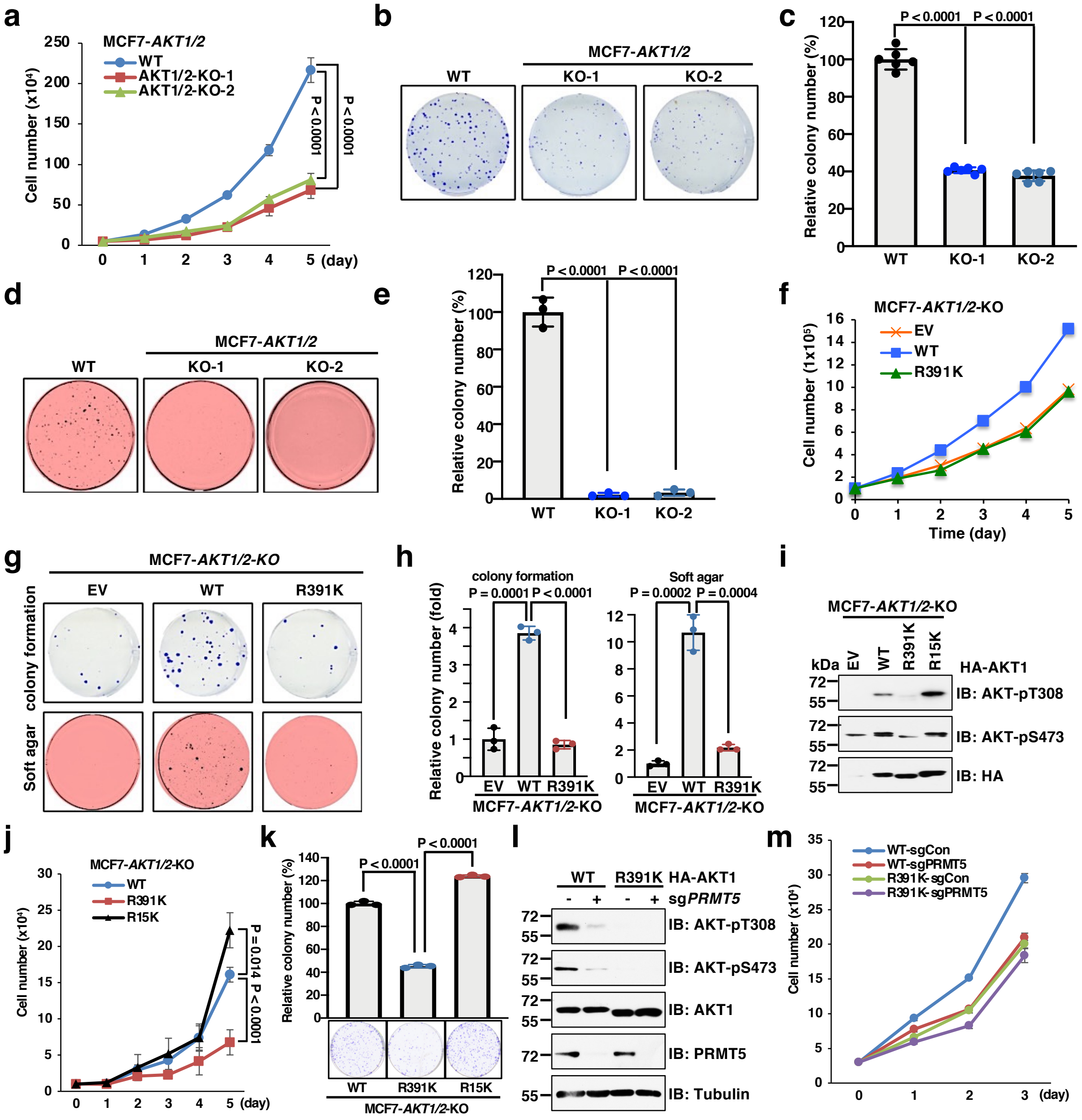

a
MCF7-AKT1/2
WT
AKT1/2-KO-1
AKT1/2-KO-2
Cell number (x10⁴)
P < 0.0001
P < 0.0001
(day)
b
MCF7-AKT1/2
WT
KO-1
KO-2
c
Relative colony number (%)
P < 0.0001
P < 0.0001
WT
KO-1
KO-2
d
MCF7-AKT1/2
WT
KO-1
KO-2
e
Relative colony number (%)
P < 0.0001
P < 0.0001
WT
KO-1
KO-2
f
MCF7-AKT1/2-KO
EV
WT
R391K
Cell number (1x10⁵)
Time (day)
g
MCF7-AKT1/2-KO
EV
WT
R391K
colony formation
Soft agar
h
colony formation
P = 0.0001
P < 0.0001
Soft agar
P = 0.0002
P = 0.0004
Relative colony number (fold)
EV
WT
R391K
MCF7-AKT1/2-KO
i
MCF7-AKT1/2-KO
kDa
EV
WT
R391K
R15K
HA-AKT1
IB: AKT-pT308
IB: AKT-pS473
IB: HA
j
MCF7-AKT1/2-KO
WT
R391K
R15K
Cell number (x10⁴)
P = 0.014
P < 0.0001
(day)
k
Relative colony number (%)
P < 0.0001
P < 0.0001
WT
R391K
R15K
MCF7-AKT1/2-KO
l
WT
R391K
HA-AKT1
sgPRMT5
IB: AKT-pT308
IB: AKT-pS473
IB: AKT1
IB: PRMT5
IB: Tubulin
m
WT-sgCon
WT-sgPRMT5
R391K-sgCon
R391K-sgPRMT5
Cell number (x10⁴)
(day)

Supplementary Fig. 8 AKT1-R391 methylation promotes cell proliferation. a MCF7-*AKT1/2*-KO cells were subjected to cell proliferation assays. Data are shown as the mean ± s.d. of n =3 independent experiments. **b-e** *AKT1/2-KO* MCF7 cells were subjected to colony formation (**b**, **c**) and soft agar assays (**d**, **e**). Data are shown as the mean ± s.d. of n =3 independent experiments. **f** MCF7-*AKT1/2-KO* cells reconstituted with AKT1-WT or R391K mutant were subjected to cell proliferation assay. Data are derived from one experiment. **g, h** MCF7-*AKT1/2-KO* cells reconstituted with AKT1-WT or R391K mutant were subjected to colony formation and soft agar assays. Data are shown as the mean ± s.d. of n =3 independent experiments. **i** IB analysis of WCL derived from MCF7-*AKT1/2*-dKO cells reconstituted with AKT1-WT, R15K or R391K mutant. Similar results were obtained in n = 3 independent experiments. **j, k** Cells generated in (**i**) were subjected to cell proliferation assays (**j**) and colony formation assays (**k**). Data are shown as the mean ± s.d. of n = 3 independent experiments. **l** IB analysis of WCL derived from DLD-1-*AKT1/2*$^{-/-}$ cells reconstituted with AKT1-WT or R391K mutant with or without *PRMT5* depletion. Similar results were obtained in n = 2 independent experiments. **m** Cells generated in (**l**) were subjected to cell proliferation assays. Data are shown as the mean ± s.d. of n = 3 independent experiments. Statistical significance was determined by two-tailed Student's t-test in **c**, **e**, **h**, **k** and two-way ANOVA in **a**, **j**, **m**. Uncropped immunoblots and statistical source data are provided in Source Data files.

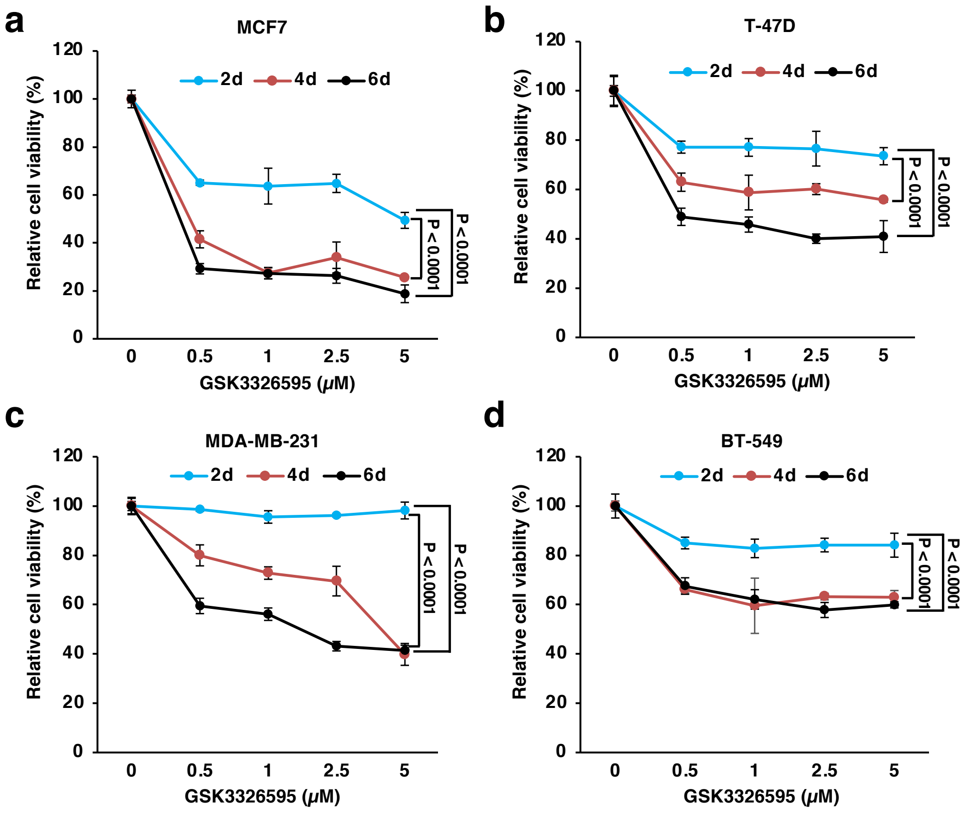

Supplementary Fig. 9 PRMT5 inhibitor decreases cell viability in a time-dependent manner. a-d MCF7, T-47D, MDA-MB-231 and BT-549 cells were treated with indicated doses of GSK3326595 for 2, 4 or 6 days and subjected to cell viability assays. Data represent the mean ± s.d. of three independent experiments. Statistical significance was determined by two-way ANOVA. Statistical source data are provided in Source Data file.

Supplementary Table 1. Sequence of sgRNAs.

PRMT1-sg-1-F	caccgTCGTCCTTCAGCATCTCCTA
PRMT1-sg-1-R	aaacTAGGAGATGCTGAAGGACGAc
PRMT1-sg-2-F	caccgCTAGGAGATGCTGAAGGACG
PRMT1-sg-2-R	aaacCGTCCTTCAGCATCTCCTAGc
PRMT2-sg-1-F	caccgCCGGCCTCACTGCACTCAGC
PRMT2-sg-1-R	aaacGCTGAGTGCAGTGAGGCCGGc
PRMT2-sg-2-F	caccgCCTGCTGAGTGCAGTGAGGC
PRMT2-sg-2-R	aaacGCCTCACTGCACTCAGCAGGc
PRMT3-sg-1-F	caccgTCAGTTTAATATTGACAGCA
PRMT3-sg-1-R	aaacTGCTGTCAATATTAAACTGAc
PRMT3-sg-2-F	caccgGACAGCATGGTTCATAAACA
PRMT3-sg-2-R	aaacTGTTTATGAACCATGCTGTCc
PRMT4-sg-1-F	caccgACGGCTGCACTCTGTCTCTC
PRMT4-sg-1-R	aaacGAGAGACAGAGTGCAGCCGTc
PRMT4-sg-2-F	caccgTGAAGGACTGCTTGCCCACA
PRMT4-sg-2-R	aaacTGTGGGCAAGCAGTCCTTCAc
PRMT5-sg-1-F	caccgGATGGAAGACAGGCATGCAG
PRMT5-sg-1-R	aaacCTGCATGCCTGTCTTCCATCc
PRMT5-sg-2-F	caccgATGAACTCCCTCTTGAAACG
PRMT5-sg-2-R	aaacCGTTTCAAGAGGGAGTTCATc
PRMT6-sg-1-F	caccgCAAGAAAAGAAAGCTTGAGT
PRMT6-sg-1-R	aaacACTCAAGCTTTCTTTTCTTGc
PRMT6-sg-2-F	caccgAAAAGAAAGCTTGAGTCGGG
PRMT6-sg-2-R	aaacCCCGACTCAAGCTTTCTTTTc
PRMT7-sg-1-F	caccgTCACGGCAGCCCGGATACCT
PRMT7-sg-1-R	aaacAGGTATCCGGGCTGCCGTGAc
PRMT7-sg-2-F	caccgAAAATACTACCAAGGTATCC
PRMT7-sg-2-R	aaacGGATACCTTGGTAGTATTTTc
PRMT9-sg-1-F	caccgGGCTTTAGGGATGAAGCAGC
PRMT9-sg-1-R	aaacGCTGCTTCATCCCTAAAGCCc
PRMT9-sg-2-F	caccgCCTTTGCATCACTGAAATCA
PRMT9-sg-2-R	aaacTGATTTCAGTGATGCAAAGGc
AKT1-sg-F	caccgGGGAGTACATCAAGACCTGG
AKT1-sg-R	aaacCCAGGTCTTGATGTACTCCCc
AKT2-sg-F	caccgCTCTTCAGCAGGAAGTACCG
AKT2-sg-R	aaacCGGTACTTCCTGCTGAAGAGc
AKT3-sg-F	caccgGAGAATATATAAAAAACTGG
AKT3-sg-R	aaacCCAGTTTTTTATATATTCTCc
