# Supplementary material for: PRMT5-mediated arginine methylation activates AKT kinase to govern tumorigenesis
Source: Nat Commun. 2021 Jun 8;12:3444. doi: 10.1038/s41467-021-23833-2 (PMC8187744; doi:10.1038/s41467-021-23833-2)
Supplement: Supplementary file 3 — Reporting Summary [file 41467_2021_23833_MOESM3_ESM.pdf]

nature research

Corresponding author(s): Wenjian Gan

Last updated by author(s): May 18, 2021

Reporting Summary

Nature Research wishes to improve the reproducibility of the work that we publish. This form provides structure for consistency and transparency in reporting. For further information on Nature Research policies, see our Editorial Policies and the Editorial Policy Checklist.

Statistics

For all statistical analyses, confirm that the following items are present in the figure legend, table legend, main text, or Methods section.

n/a	Confirmed	
☐	☒	The exact sample size (*n*) for each experimental group/condition, given as a discrete number and unit of measurement
☐	☒	A statement on whether measurements were taken from distinct samples or whether the same sample was measured repeatedly
☐	☒	The statistical test(s) used AND whether they are one- or two-sided<br>*Only common tests should be described solely by name; describe more complex techniques in the Methods section.*
☒	☐	A description of all covariates tested
☒	☐	A description of any assumptions or corrections, such as tests of normality and adjustment for multiple comparisons
☐	☒	A full description of the statistical parameters including central tendency (e.g. means) or other basic estimates (e.g. regression coefficient) AND variation (e.g. standard deviation) or associated estimates of uncertainty (e.g. confidence intervals)
☐	☒	For null hypothesis testing, the test statistic (e.g. *F*, *t*, *r*) with confidence intervals, effect sizes, degrees of freedom and *P* value noted<br>*Give P values as exact values whenever suitable.*
☒	☐	For Bayesian analysis, information on the choice of priors and Markov chain Monte Carlo settings
☒	☐	For hierarchical and complex designs, identification of the appropriate level for tests and full reporting of outcomes
☒	☐	Estimates of effect sizes (e.g. Cohen's *d*, Pearson's *r*), indicating how they were calculated

Our web collection on statistics for biologists contains articles on many of the points above.

Software and code

Policy information about availability of computer code

Data collection	No software was used
Data analysis	We used the GraphPad Prism 9, Excel (version 16.48), imageJ (1.53a) and MaxQuant (v1.6.17.0) to generate the graph figures and perform significance analysis.

For manuscripts utilizing custom algorithms or software that are central to the research but not yet described in published literature, software must be made available to editors and reviewers. We strongly encourage code deposition in a community repository (e.g. GitHub). See the Nature Research guidelines for submitting code & software for further information.

Data

Policy information about availability of data

All manuscripts must include a data availability statement. This statement should provide the following information, where applicable:

- Accession codes, unique identifiers, or web links for publicly available datasets
- A list of figures that have associated raw data
- A description of any restrictions on data availability

All relevant data are included in the paper and supplementary information files. Uncropped images for immunoblots and statistical source data are provided with this paper. The mass spectrometry raw data are available via ProteomeXchange with identifier PXD026071 https://www.ebi.ac.uk/pride/archive/projects/PXD026071.

Field-specific reporting

Please select the one below that is the best fit for your research. If you are not sure, read the appropriate sections before making your selection.

☒ Life sciences ☐ Behavioural & social sciences ☐ Ecological, evolutionary & environmental sciences

For a reference copy of the document with all sections, see nature.com/documents/nr-reporting-summary-flat.pdf

Life sciences study design

All studies must disclose on these points even when the disclosure is negative.

Sample size	No sample size calculation was performed for this study. Sample size for animal study is 4-7 mice per group, which obtained 7-9 tumors.
Data exclusions	No data was excluded from the experiments.
Replication	All biochemical experiments were repeated at least twice and obtained similar results for all replications.
Randomization	For the mouse xenograft assay in Fig. 2d, 2e and 6g-i, null mice (from Taconic Bioscience) were randomized into four or two groups and injected with indicated cell lines. Randomization was not relevant to other experiments in the study.
Blinding	The investigators were not blinded to group allocation during data collection and/or analysis because we measured the value of tumor size or mouse body/organ weight and did not have drug treatment for these experiments.

Reporting for specific materials, systems and methods

We require information from authors about some types of materials, experimental systems and methods used in many studies. Here, indicate whether each material, system or method listed is relevant to your study. If you are not sure if a list item applies to your research, read the appropriate section before selecting a response.

Materials & experimental systems

n/a	Involved in the study	
☐	☒	Antibodies
☐	☒	Eukaryotic cell lines
☒	☐	Palaeontology and archaeology
☐	☒	Animals and other organisms
☒	☐	Human research participants
☒	☐	Clinical data
☒	☐	Dual use research of concern

Methods

n/a	Involved in the study	
☒	☐	ChIP-seq
☒	☐	Flow cytometry
☒	☐	MRI-based neuroimaging

Antibodies

Antibodies used	Anti-AKT-pT308 antibody (13038, 1:1000), anti-AKT-pS473 antibody (4060, 1:3000), anti-AKT1 antibody (2938, 1:3000), anti-AKT pan antibody (4685, 1:3000), anti-pGSK3β antibody (5558, 1:5000), anti-GSK3β antibody (12456, 1:5000), anti-pFOXO antibody (2599, 1;1000), anti-PRMT1 (2449, 1:1000), anti-PRMT4 antibody (12495, 1:1000), anti-PRMT5 antibody (79998, 1:1000), anti-S6K1 antibody (9202, 1:2000), anti-E-cadherin antibody (3195, 1:3000), anti-PDK1 antibody (13037, 1:1000), anti-Ki-67 antibody (9027, 1:800 for IHC), anti-Myc-tag rabbit antibody (2278, 1:1000), anti-GST rabbit antibody (2625, 1:2000), anti-HA rabbit antibody (3724, 1:2000) and anti-Sdme-RG antibody (13222, 1:1000) were purchased from Cell Signaling Technology. Anti-FOXO3A antibody (10849-1-AP, 1:2000), anti-PRMT5 antibody (18436-1-AP, 1:1000 for IHC) and anti-Tubulin antibody (66240-1-lg, 1:10000) were purchased from Proteintech. Anti-sym11 antibody (07-413, 1:1000), anti-FLAG rabbit antibody (F7425, 1:2000), anti-FLAG mouse antibody (F3165, 1:2000), peroxidase-conjugated anti-mouse secondary antibody (A4416, 1:3000) and anti-rabbit secondary antibody (A4914, 1:3000) were obtained from Sigma. Monoclonal anti-HA antibody (901503, 1:3000) was purchased from Biolegend. Anti-PRMT2 antibody (720141, 1:500) was purchased from Thermo Fisher. Anti-PRMT3 antibody (ab191562, 1:10000) was purchased from Abcam. Anti-PRMT6 antibody (sc-271744, 1:2000), was purchased from Santa Cruz Biotechnology. Anti-PRMT7 antibody (A12159, 1:2000) was purchased from ABclonal. Anti-PRMT9 antibody (A304-189A, 1:1000) was purchased from Bethyl Laboratories. Anti-R391-me2s antibody (1:1000 for IHC). Goat anti-Rabbit IgG Alexa Fluor 488 (Invitrogen, #A-11008, 1:400). Anti-AKT1-R391-me2s (1;1000) and anti-AKT1-R391-control antibody (1;1000) were generated by ABclonal.
Validation	The AKT1-R391-me2s antibody was validated by western blot and IHC analysis of human AKT-R391 methylation in Fig. 3f-3i and Supplementary Fig. 3m-3p.<br>All other primary antibodies used in this study have been validated and detailed information could be found on the website from manufactures as listed below.<br>anti-AKT-pT308 antibody (13038), https://www.cellsignal.com//products/primary-antibodies/phospho-akt-thr308-d25e6-xp-rabbit-mab/13038.

anti-AKT-pS473 antibody (4060), https://www.cellsignal.com//products/primary-antibodies/phospho-akt-ser473-d9e-xp-rabbit-mab/4060

anti-AKT1 antibody (2938), https://www.cellsignal.com//products/primary-antibodies/akt1-c73h10-rabbit-mab/2938

anti-AKT pan antibody (4685), https://www.cellsignal.com//products/primary-antibodies/akt-pan-11e7-rabbit-mab/4685

anti-pGSK3β antibody (5558), https://www.cellsignal.com//products/primary-antibodies/phospho-gsk-3b-ser9-d85e12-xp-rabbit-mab/5558

anti-GSK3β antibody (12456), https://www.cellsignal.com//products/primary-antibodies/gsk-3b-d5c5z-xp-rabbit-mab/12456

anti-pFOXO antibody (2599), https://www.cellsignal.com//products/primary-antibodies/phospho-foxo1-thr24-foxo3a-thr32-foxo4-thr28-4g6-rabbit-mab/2599

anti-PRMT1 (2449), https://www.cellsignal.com//products/primary-antibodies/prmt1-a33-antibody/2449

anti-PRMT4 antibody (12495), https://www.cellsignal.com//products/primary-antibodies/prmt4-carm1-3h2-mouse-mab/12495

anti-PRMT5 antibody (79998), https://www.cellsignal.com//products/primary-antibodies/prmt5-d5p2t-rabbit-mab/79998

anti-S6K1 antibody (9202), https://www.cellsignal.com//products/primary-antibodies/p70-s6-kinase-antibody/9202

anti-E-cadherin antibody (3195), https://www.cellsignal.com//products/primary-antibodies/e-cadherin-24e10-rabbit-mab/3195

anti-PDK1 antibody (13037), https://www.cellsignal.com//products/primary-antibodies/pdk1-d4q4d-rabbit-mab/13037

anti-Ki-67 antibody (9027), https://www.cellsignal.com//products/primary-antibodies/ki-67-d2h10-rabbit-mab-ihc-specific/9027

anti-Myc-tag rabbit antibody (2278), https://www.cellsignal.com//products/primary-antibodies/myc-tag-71d10-rabbit-mab/2278

anti-GST rabbit antibody (2625), https://www.cellsignal.com//products/primary-antibodies/gst-91g1-rabbit-mab/2625

anti-HA rabbit antibody (3724), https://www.cellsignal.com//products/primary-antibodies/ha-tag-c29f4-rabbit-mab/3724

anti-Sdme-RG antibody (13222), https://www.cellsignal.com//products/primary-antibodies/symmetric-di-methyl-arginine-motif-sdme-rg-multimab-rabbit-mab-mix/13222

anti-FOXO3A antibody (10849-1-AP), https://www.ptglab.com/products/FOXO3A-Antibody-10849-1-AP.htm

anti-Tubulin antibody (66240-1-lg), https://www.ptglab.com/products/Tubulin-beta-Antibody-66240-1-Ig.htm

anti-PRMT5 antibody (18436-1-AP), https://www.ptglab.com/products/PRMT5-Antibody-18436-1-AP.htm

Anti-sym11 antibody (07-413), https://www.sigmaaldrich.com/catalog/product/mm/07413?lang=en&region=US

anti-FLAG rabbit antibody (F7425), https://www.sigmaaldrich.com/catalog/product/sigma/f7425?lang=en&region=US

anti-FLAG mouse antibody (F3165), https://www.sigmaaldrich.com/catalog/product/sigma/f3165?lang=en&region=US

anti-HA monoclonal antibody (901503), https://www.biolegend.com/en-us/products/purified-anti-ha-11-epitope-tag-antibody-11374

Anti-PRMT2 antibody (720141), https://www.thermofisher.com/antibody/product/PRMT2-Antibody-Polyclonal/720141

Anti-PRMT3 antibody (ab191562), https://www.abcam.com/prmt3-antibody-epr13279-ab191562.html

Anti-PRMT6 antibody (sc-271744), https://www.scbt.com/p/prmt6-antibody-d-5?requestFrom=search

Anti-PRMT7 antibody (A12159), https://abclonal.com/catalog-antibodies/PRMT7PolyclonalAntibody/A12159

Anti-PRMT9 antibody (A304-189A), https://www.bethyl.com/product/A304-189A?referrer=search

Eukaryotic cell lines

Policy information about cell lines

Cell line source(s)	MCF7, MDA-MB-231, HEK293, HEK293T, HeLa, DLD-1, T-47D, HCC1937, BT-549, MDA-MB-468, MDA-MB-436 cells were obtained from ATCC. The Tsc2 mouse embryonic fibroblasts (MEFs) were obtained from Dr. Branden Manning group at Harvard T.H. Chan School Of Public Health.
Authentication	No.
Mycoplasma contamination	Yes. Cell lines used in this study were routinely tested to be negative for mycoplasma.

Commonly misidentified lines (See ICLAC register)	No.

Animals and other organisms

Policy information about studies involving animals; ARRIVE guidelines recommended for reporting animal research

Laboratory animals	5-week-old female nude mice (The Jackson Laboratory) were used for the mouse xenograft assay in Fig. 2d, 2e and 6g-i.
Wild animals	The study does not use wild animals.
Field-collected samples	No.
Ethics oversight	The MUSC Institutional Animal Care and Use Committee.

Note that full information on the approval of the study protocol must also be provided in the manuscript.
