# Supplementary material for: PRMT5-mediated arginine methylation activates AKT kinase to govern tumorigenesis
Source: Nat Commun. 2021 Jun 8;12:3444. doi: 10.1038/s41467-021-23833-2 (PMC8187744; doi:10.1038/s41467-021-23833-2)

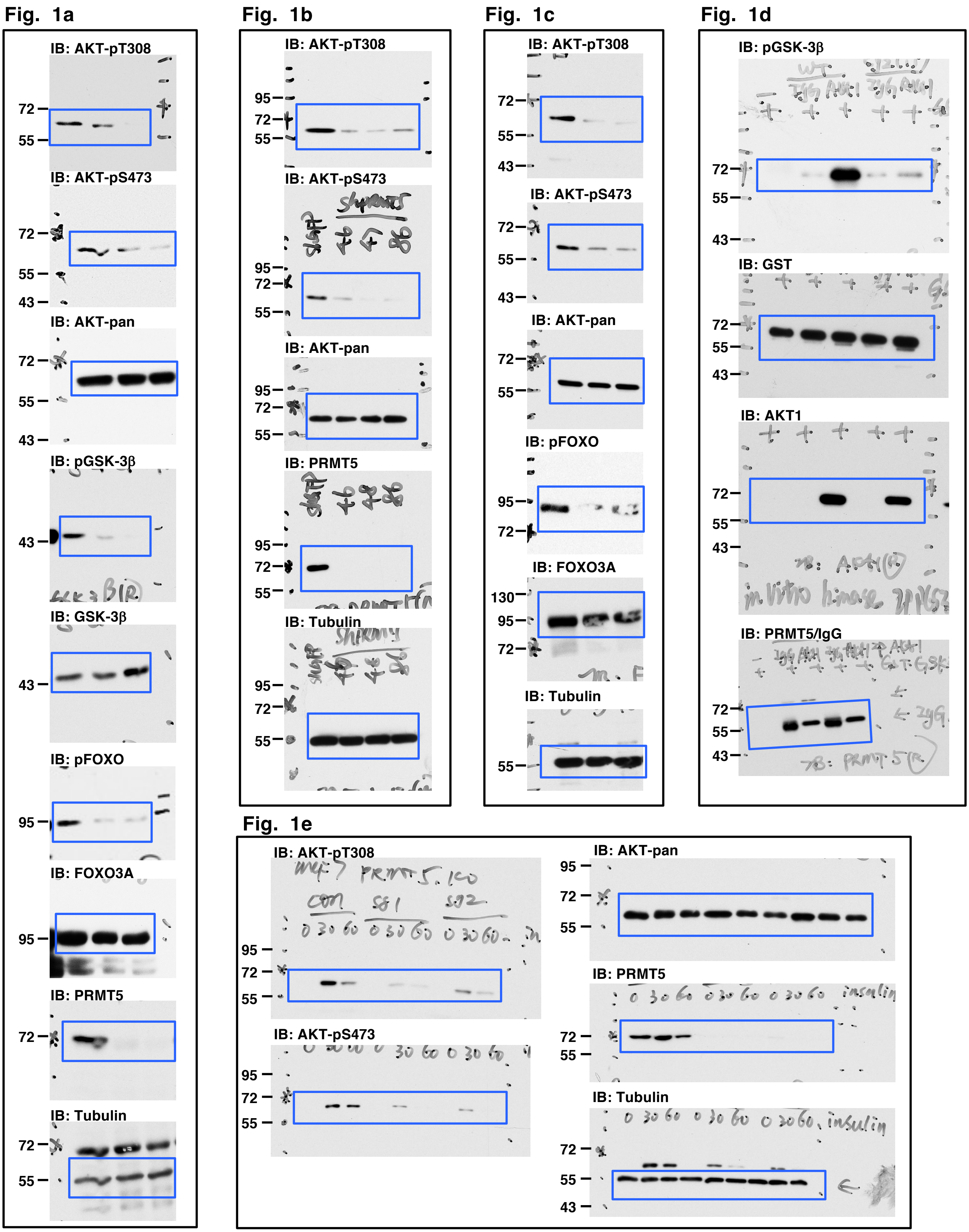
Fig. 1a
IB: AKT-pT308
IB: AKT-pS473
IB: AKT-pan
IB: pGSK-3β
IB: GSK-3β
IB: pFOXO
IB: FOXO3A
IB: PRMT5
IB: Tubulin
Fig. 1b
IB: AKT-pT308
IB: AKT-pS473
IB: AKT-pan
IB: PRMT5
IB: Tubulin
Fig. 1c
IB: AKT-pT308
IB: AKT-pS473
IB: AKT-pan
IB: pFOXO
IB: FOXO3A
IB: Tubulin
Fig. 1d
IB: pGSK-3β
IB: GST
IB: AKT1
IB: PRMT5/IgG
Fig. 1e
IB: AKT-pT308
IB: AKT-pS473
IB: AKT-pan
IB: PRMT5
IB: Tubulin
130
95
72
55
43

Fig. 2a

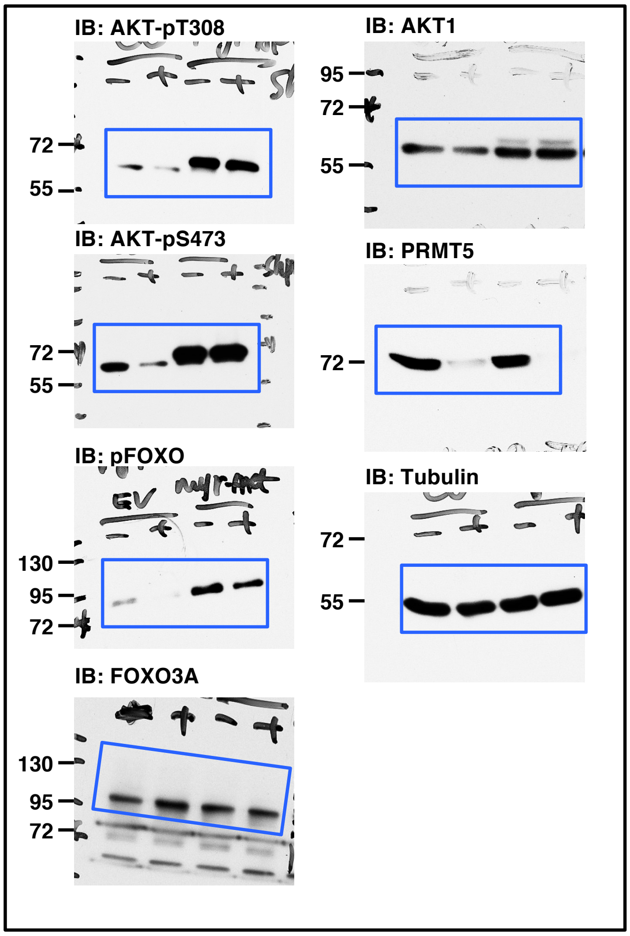

IB: AKT-pT308
72
55
IB: AKT-pS473
72
55
IB: pFOXO
EV
myr-Akt
130
95
72
IB: FOXO3A
130
95
72
IB: AKT1
95
72
55
IB: PRMT5
72
IB: Tubulin
72
55

Fig. 3a

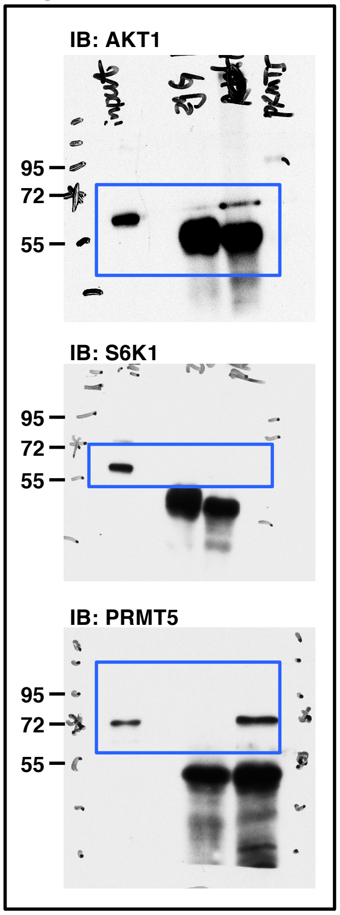

Fig. 3b

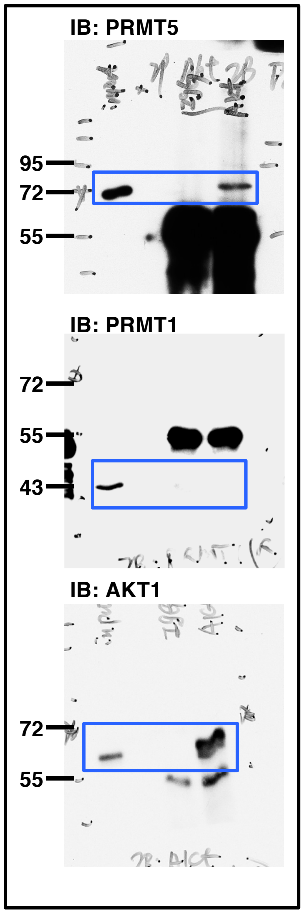

Fig. 3c

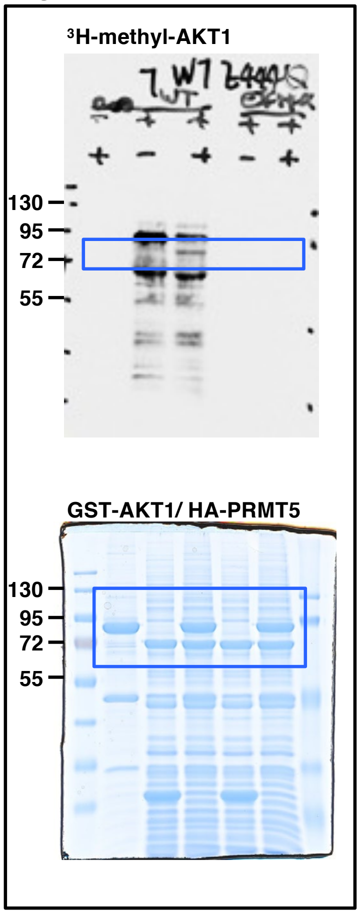

Fig. 3e

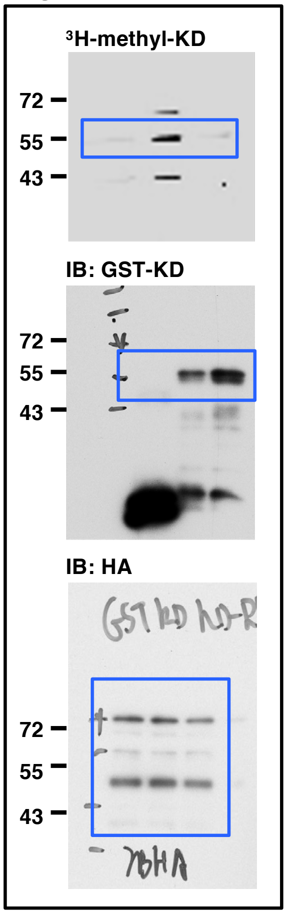

Fig. 3f

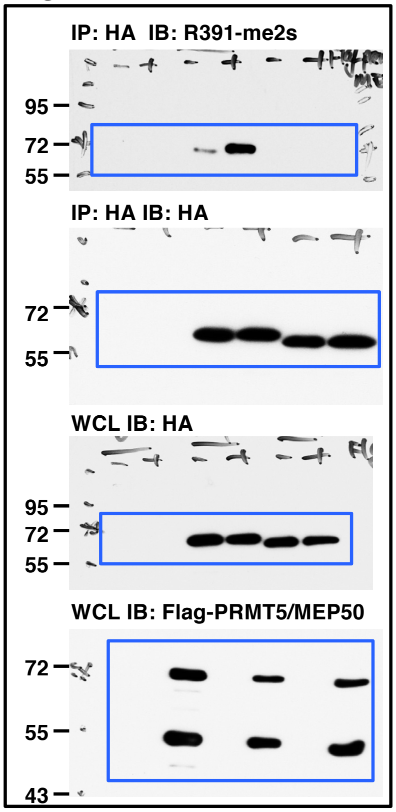

Fig. 3g

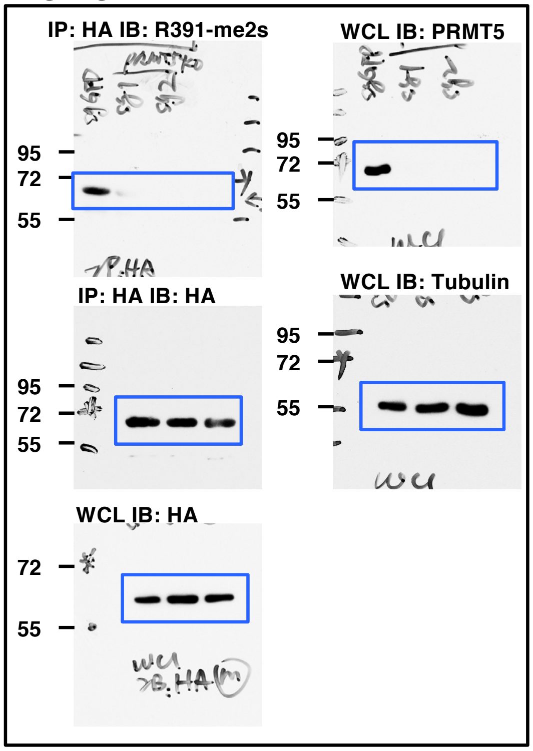

Fig. 4a

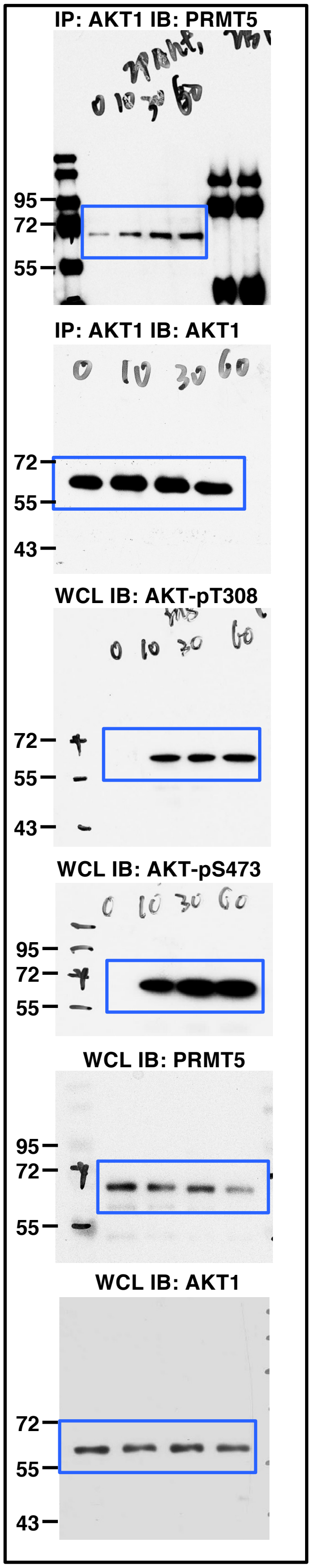

Fig. 4b

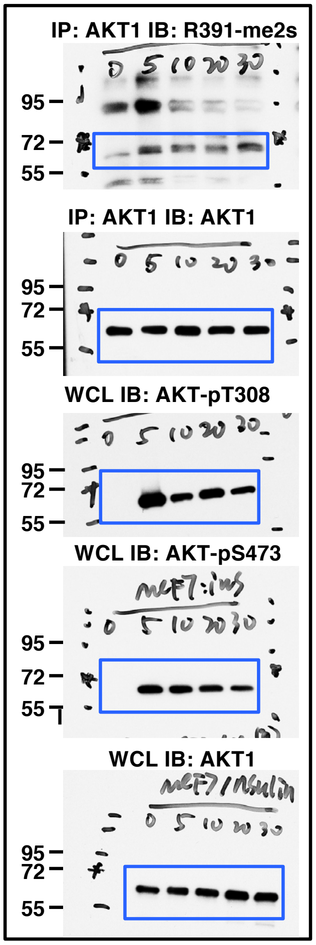

Fig. 4c

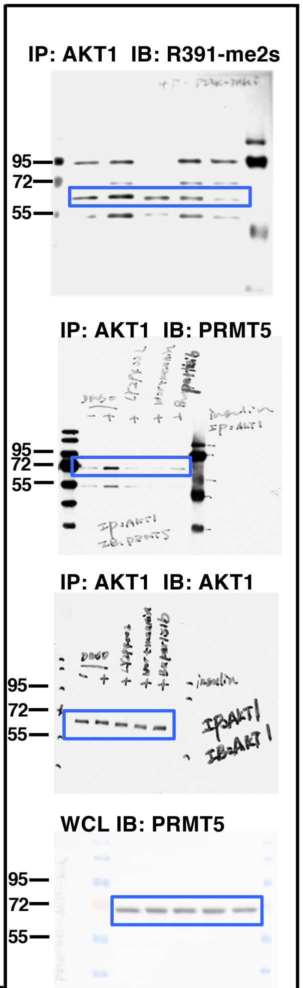

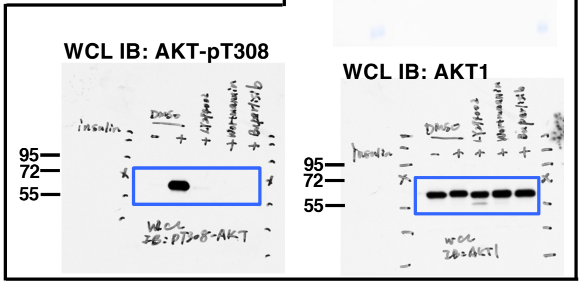

Fig. 4d

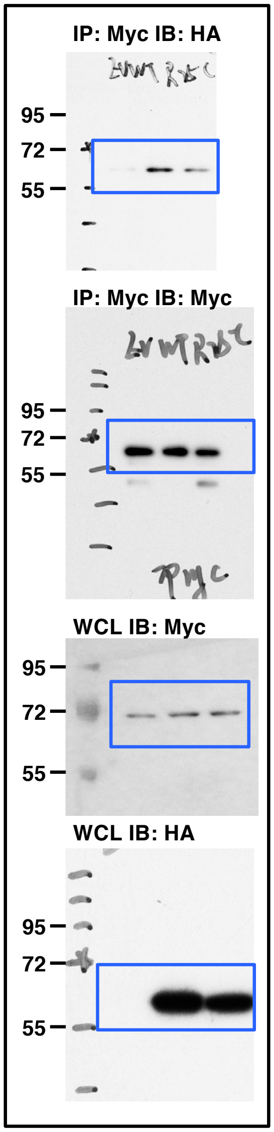

Fig. 4e

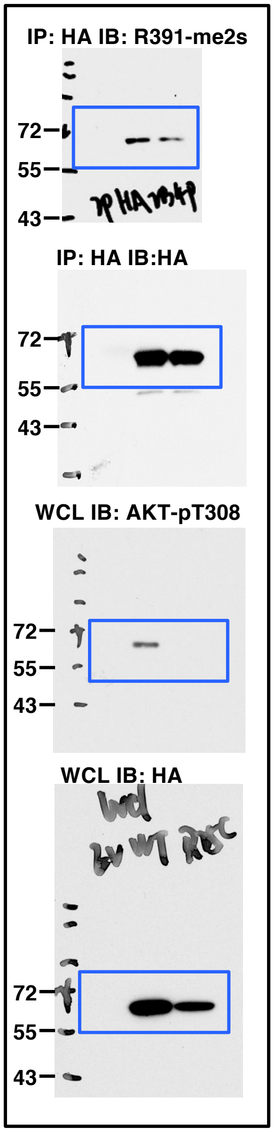

Fig. 4f

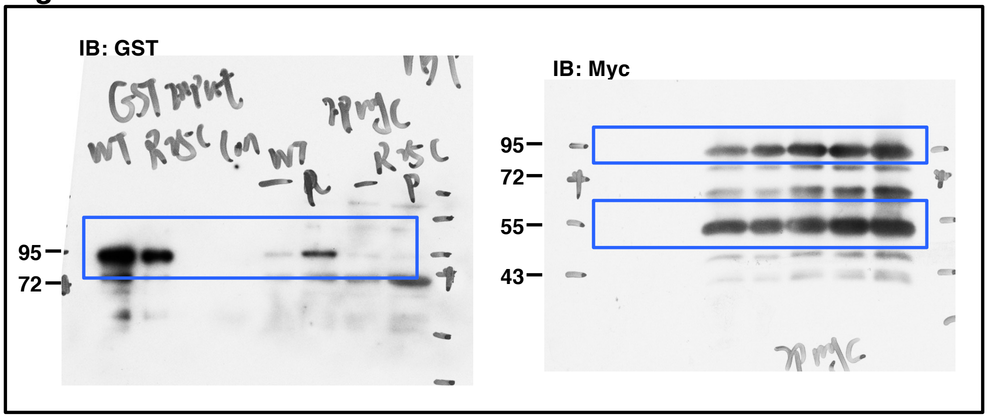

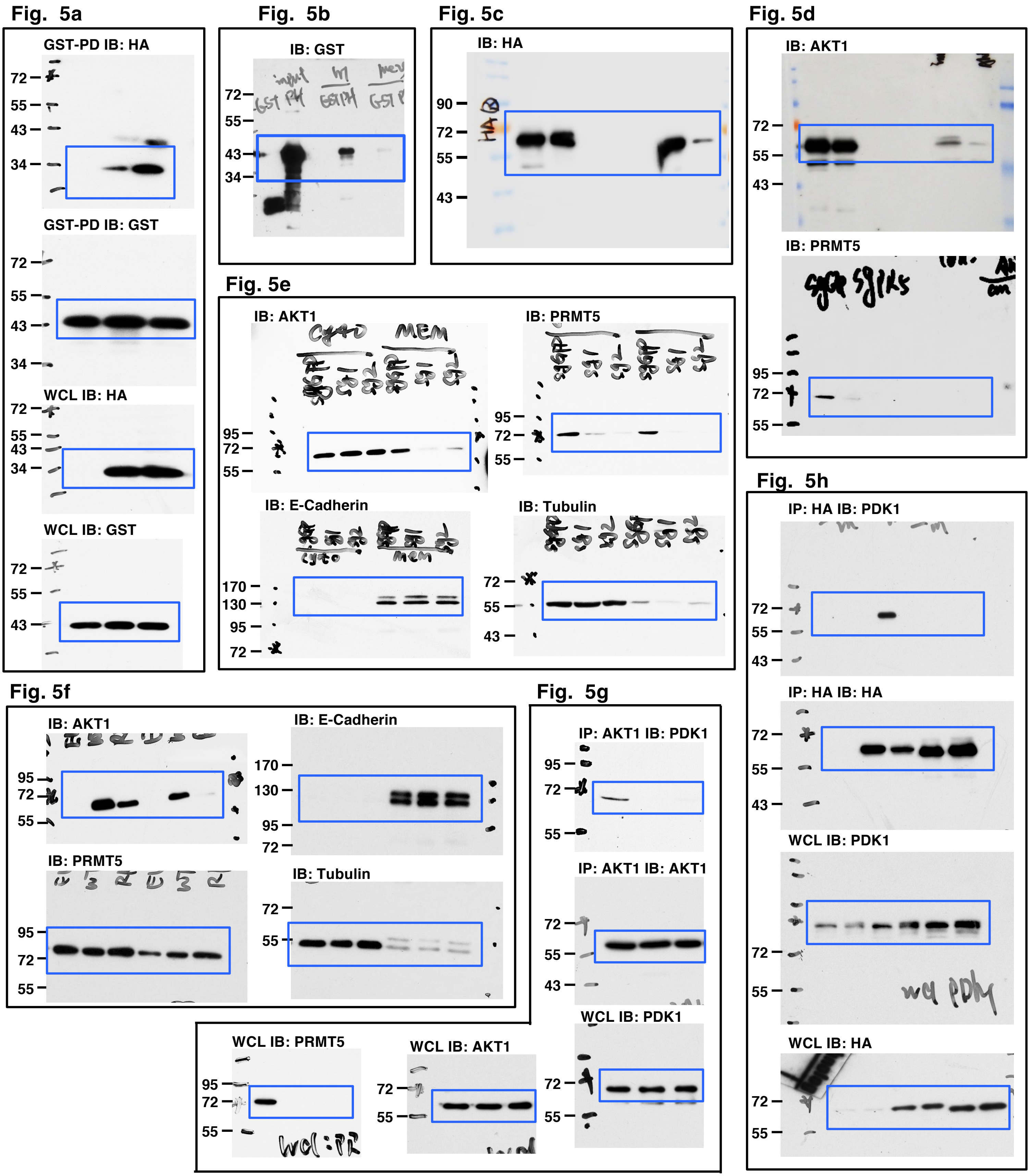

Fig. 5a
GST-PD IB: HA
GST-PD IB: GST
WCL IB: HA
WCL IB: GST
Fig. 5b
IB: GST
Fig. 5c
IB: HA
Fig. 5d
IB: AKT1
IB: PRMT5
Fig. 5e
IB: AKT1
IB: PRMT5
IB: E-Cadherin
IB: Tubulin
Fig. 5f
IB: AKT1
IB: E-Cadherin
IB: PRMT5
IB: Tubulin
Fig. 5g
IP: AKT1 IB: PDK1
IP: AKT1 IB: AKT1
WCL IB: PDK1
WCL IB: PRMT5
WCL IB: AKT1
Fig. 5h
IP: HA IB: PDK1
IP: HA IB: HA
WCL IB: PDK1
WCL IB: HA

Fig. 6a

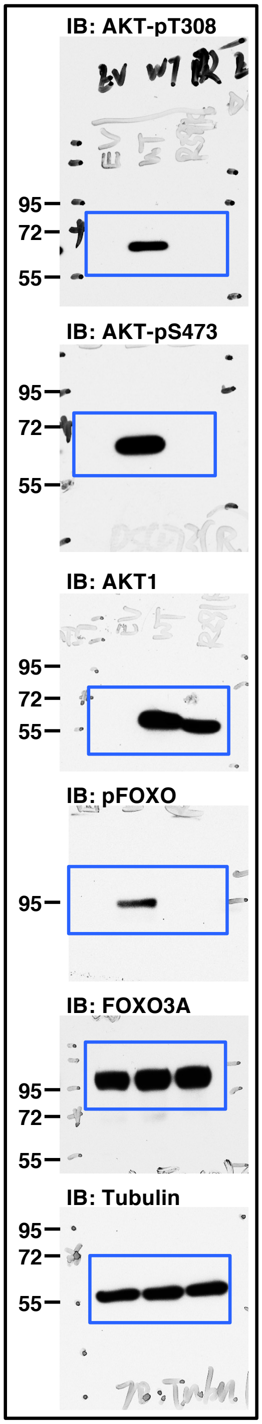

Fig. 6b

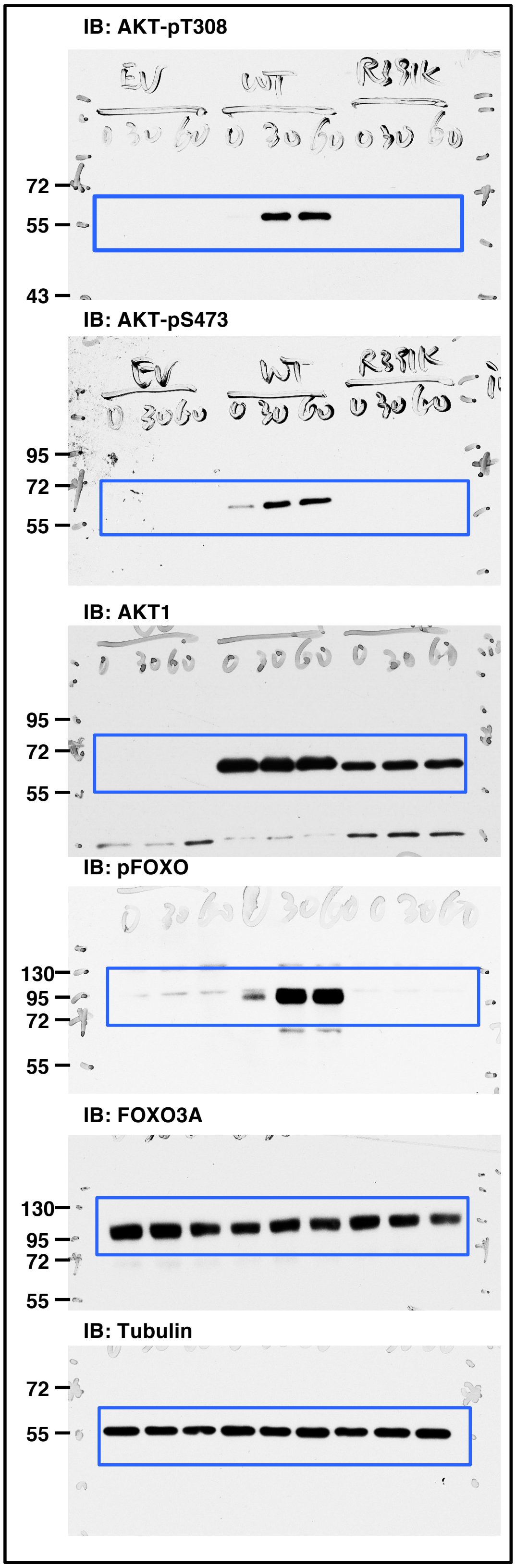

Fig. 6c

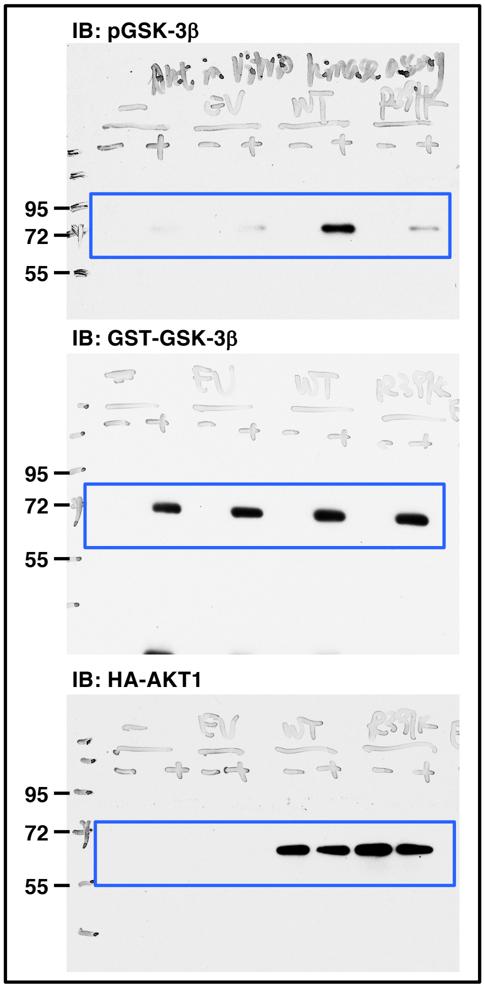

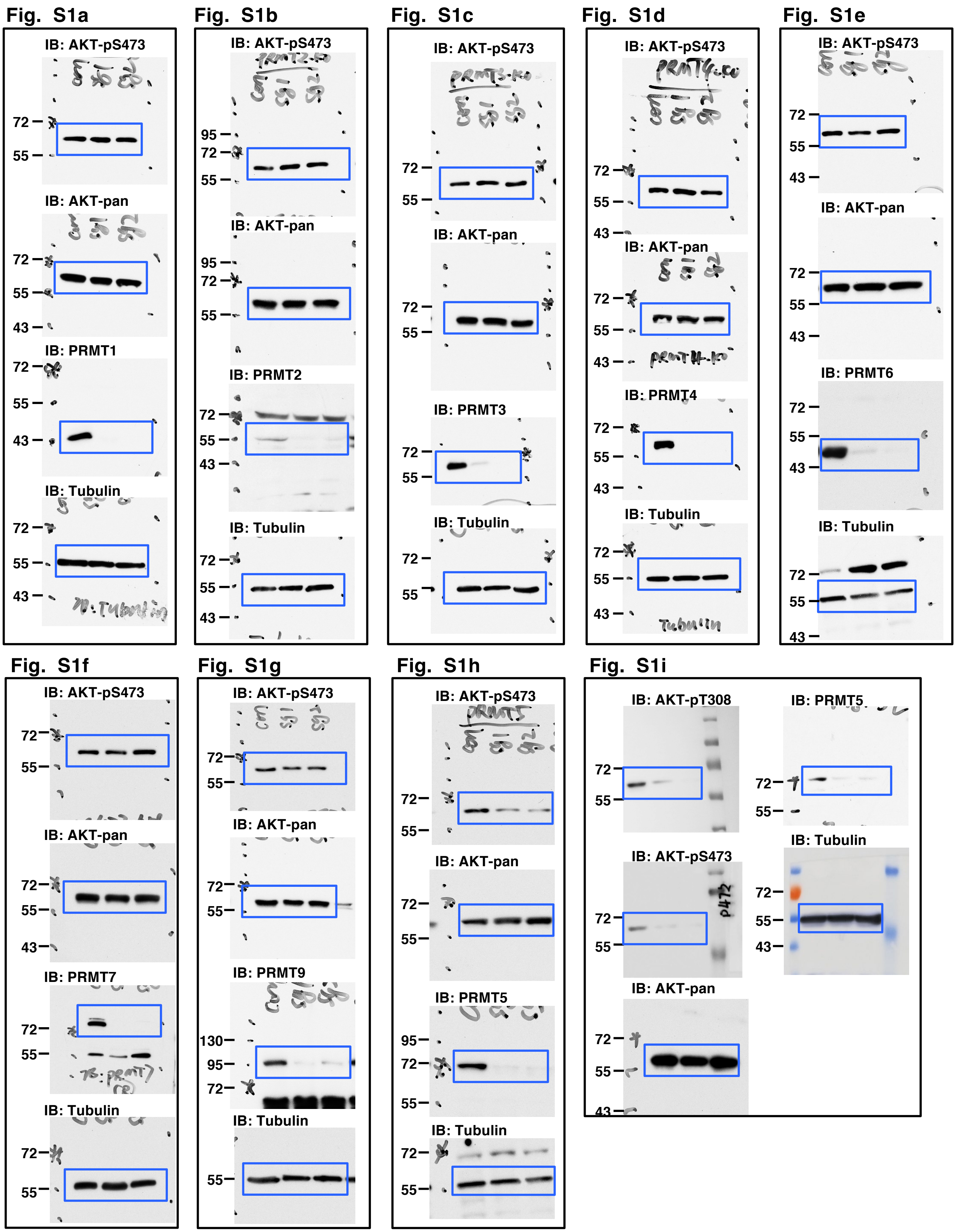

Fig. S1a
IB: AKT-pS473
72
55
IB: AKT-pan
72
55
43
IB: PRMT1
72
55
43
IB: Tubulin
72
55
43
Fig. S1b
IB: AKT-pS473
95
72
55
IB: AKT-pan
95
72
55
IB: PRMT2
72
55
43
IB: Tubulin
72
55
43
Fig. S1c
IB: AKT-pS473
72
55
IB: AKT-pan
72
55
IB: PRMT3
72
55
IB: Tubulin
72
55
Fig. S1d
IB: AKT-pS473
72
55
43
IB: AKT-pan
72
55
43
IB: PRMT4
72
55
43
IB: Tubulin
72
55
43
Fig. S1e
IB: AKT-pS473
72
55
43
IB: AKT-pan
72
55
43
IB: PRMT6
72
55
43
IB: Tubulin
72
55
43
Fig. S1f
IB: AKT-pS473
72
55
IB: AKT-pan
72
55
43
IB: PRMT7
72
55
IB: Tubulin
72
55
Fig. S1g
IB: AKT-pS473
72
55
IB: AKT-pan
72
55
IB: PRMT9
130
95
72
IB: Tubulin
55
Fig. S1h
IB: AKT-pS473
72
55
IB: AKT-pan
72
55
IB: PRMT5
95
72
55
IB: Tubulin
72
55
Fig. S1i
IB: AKT-pT308
72
55
IB: PRMT5
72
55
IB: AKT-pS473
72
55
IB: Tubulin
72
55
43
IB: AKT-pan
72
55
43

Fig. S1j

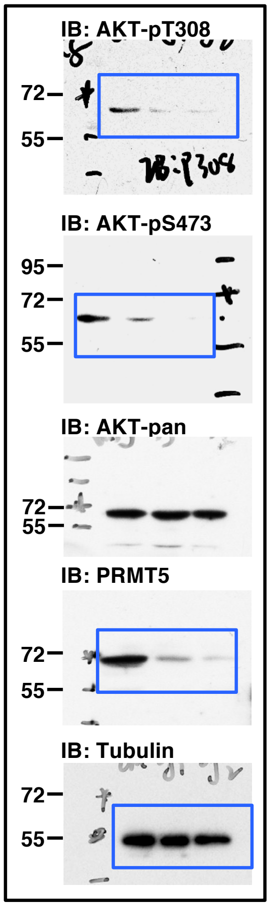

Fig. S1k

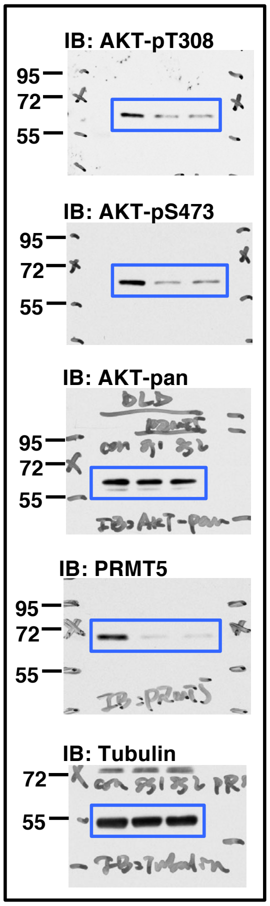

Fig. S1l

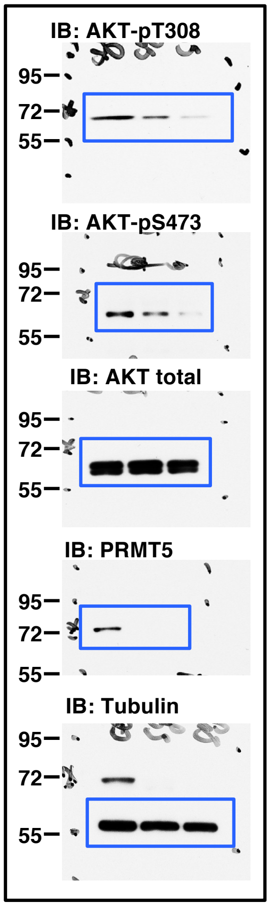

Fig. S1m

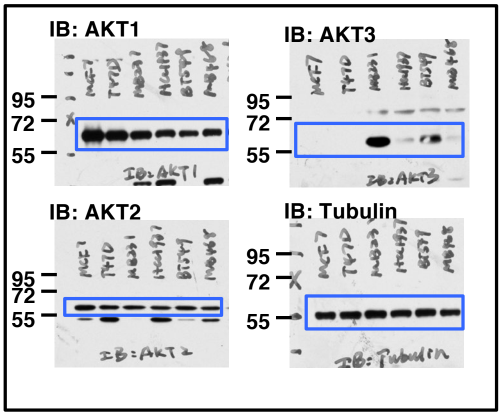

Fig. S1n

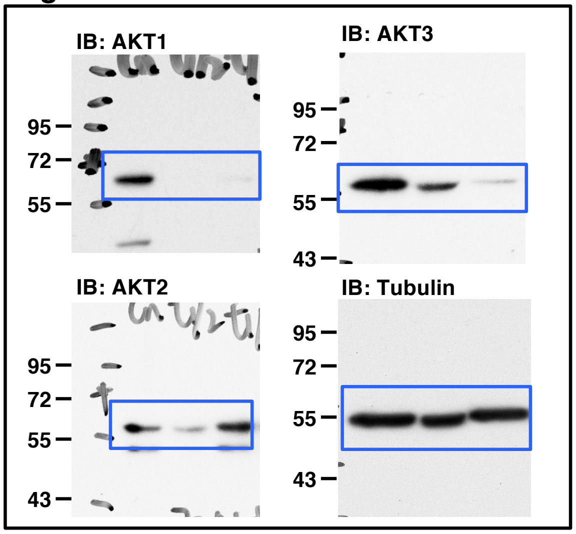

Fig. S1o

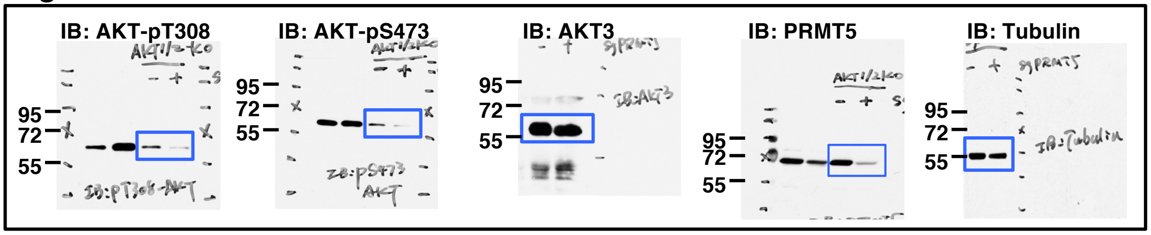

Fig. S1p

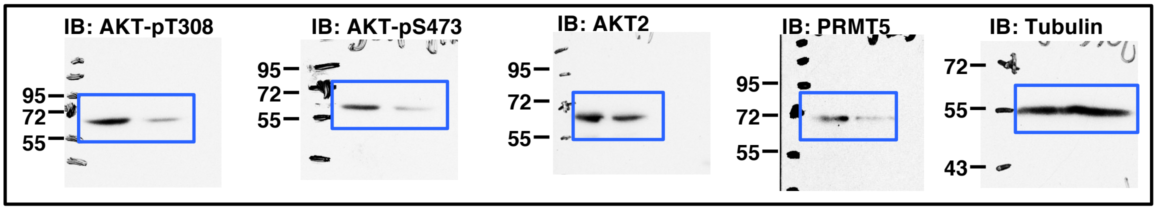

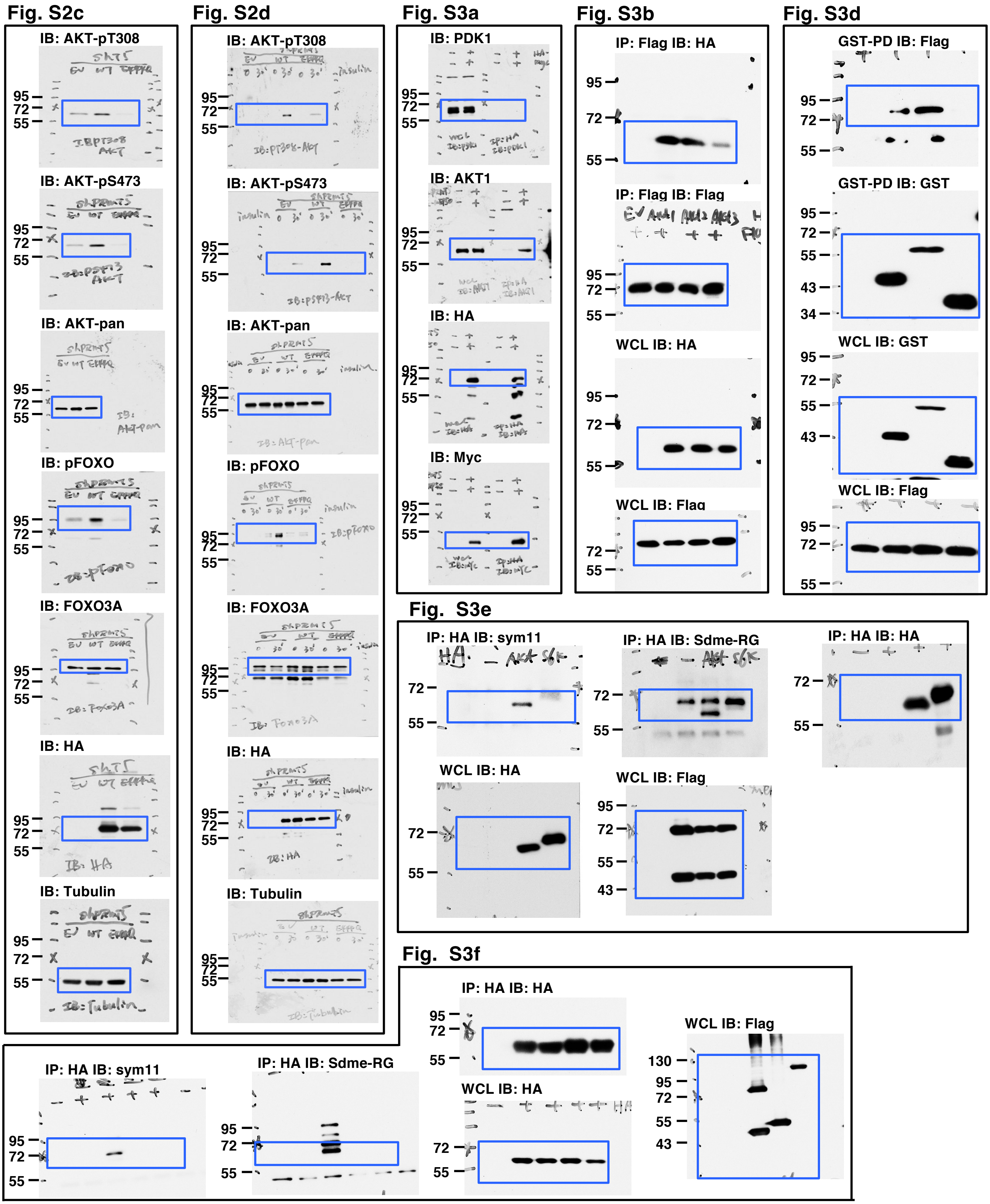
Fig. S2c
IB: AKT-pT308
IB: AKT-pS473
IB: AKT-pan
IB: pFOXO
IB: FOXO3A
IB: HA
IB: Tubulin
Fig. S2d
IB: AKT-pT308
IB: AKT-pS473
IB: AKT-pan
IB: pFOXO
IB: FOXO3A
IB: HA
IB: Tubulin
Fig. S3a
IB: PDK1
IB: AKT1
IB: HA
IB: Myc
Fig. S3b
IP: Flag IB: HA
IP: Flag IB: Flag
WCL IB: HA
WCL IB: Flag
Fig. S3d
GST-PD IB: Flag
GST-PD IB: GST
WCL IB: GST
WCL IB: Flag
Fig. S3e
IP: HA IB: sym11
IP: HA IB: Sdme-RG
IP: HA IB: HA
WCL IB: HA
WCL IB: Flag
Fig. S3f
IP: HA IB: HA
WCL IB: HA
WCL IB: Flag
IP: HA IB: sym11
IP: HA IB: Sdme-RG
130
95
72
55
43
34

Fig. S3g

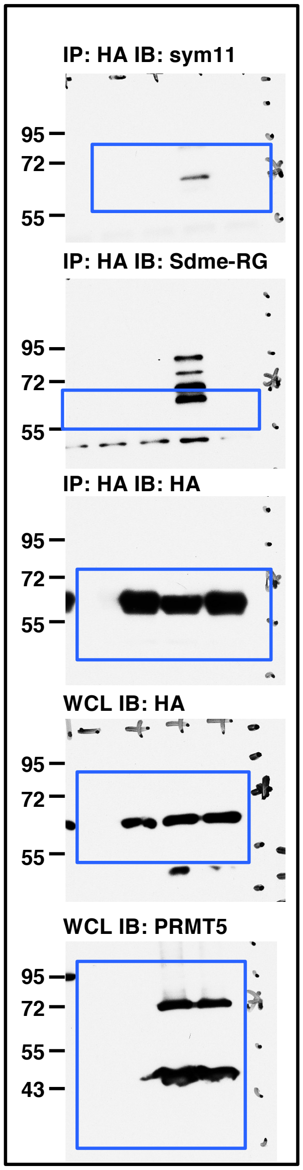

Fig. S3h

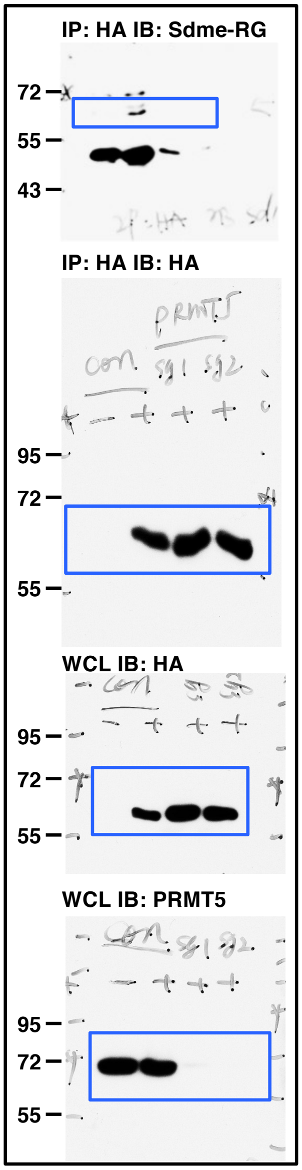

Fig. S3j

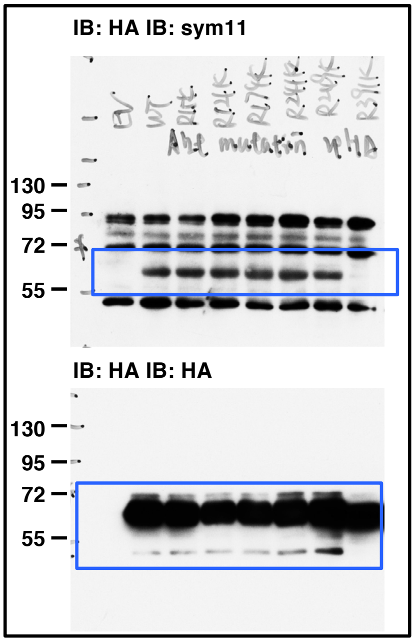

Fig. S3k

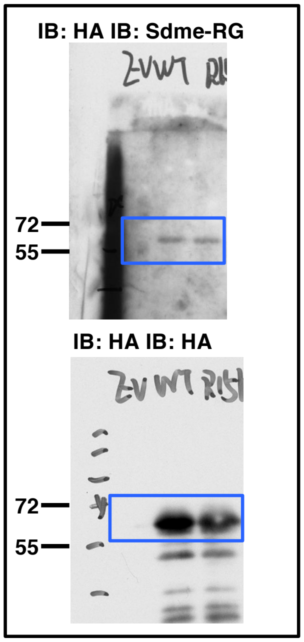

Fig. S3n

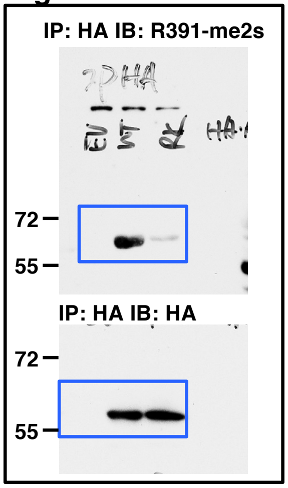

Fig. S3o

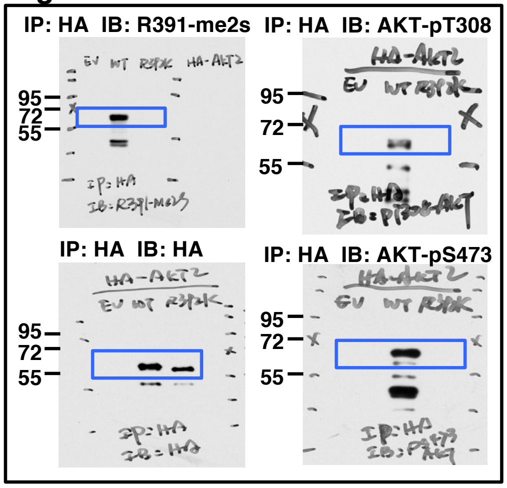

Fig. S3p

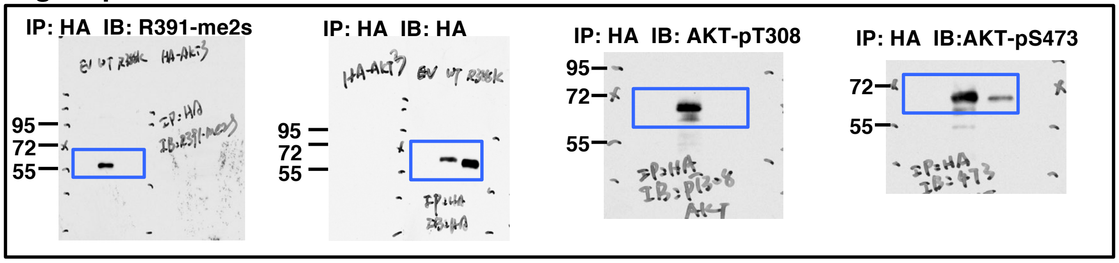

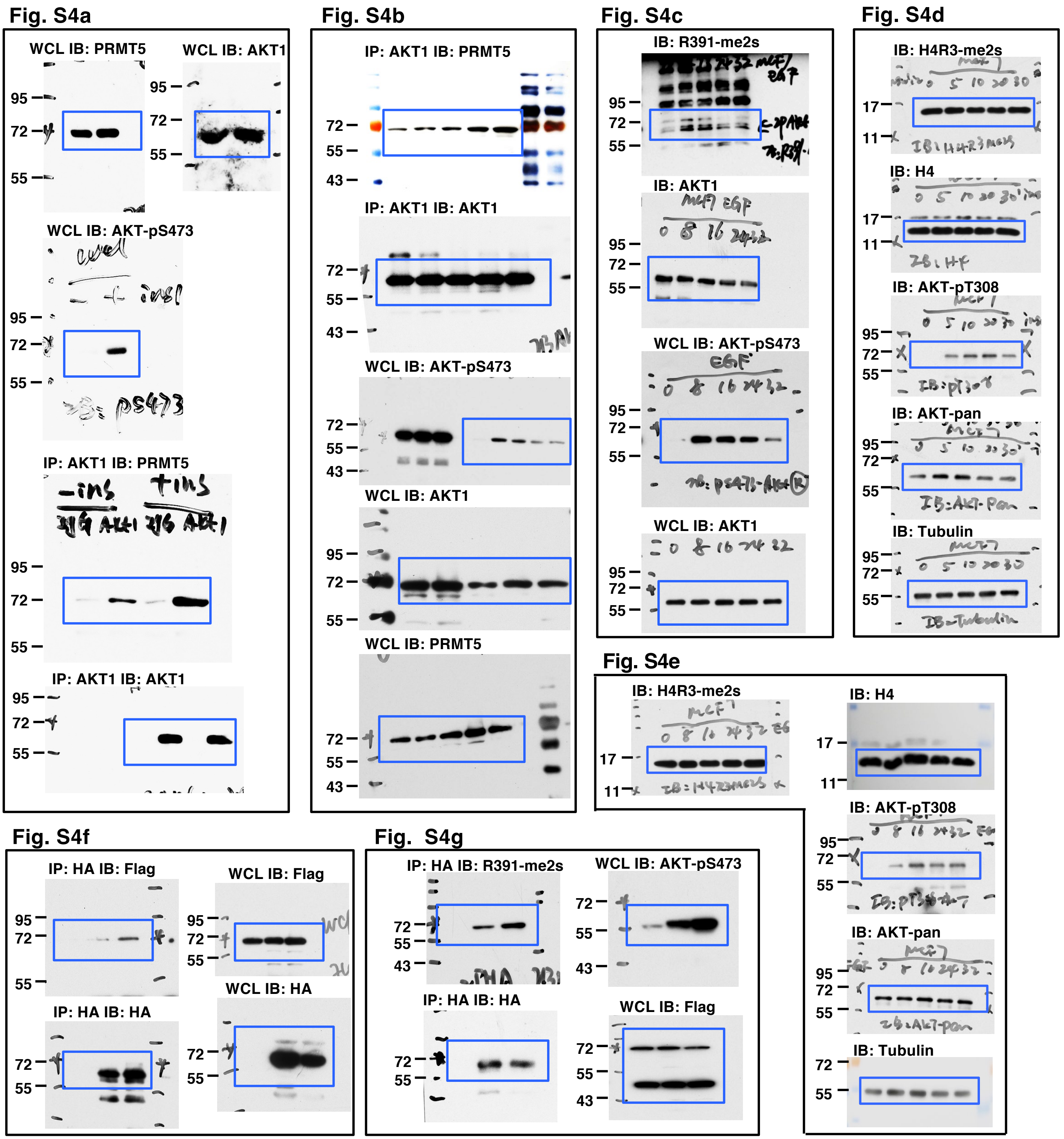

Fig. S4a
WCL IB: PRMT5
WCL IB: AKT1
WCL IB: AKT-pS473
IP: AKT1 IB: PRMT5
IP: AKT1 IB: AKT1
Fig. S4b
IP: AKT1 IB: PRMT5
IP: AKT1 IB: AKT1
WCL IB: AKT-pS473
WCL IB: AKT1
WCL IB: PRMT5
Fig. S4c
IB: R391-me2s
IB: AKT1
WCL IB: AKT-pS473
WCL IB: AKT1
Fig. S4d
IB: H4R3-me2s
IB: H4
IB: AKT-pT308
IB: AKT-pan
IB: Tubulin
Fig. S4e
IB: H4R3-me2s
IB: H4
IB: AKT-pT308
IB: AKT-pan
IB: Tubulin
Fig. S4f
IP: HA IB: Flag
WCL IB: Flag
WCL IB: HA
IP: HA IB: HA
Fig. S4g
IP: HA IB: R391-me2s
WCL IB: AKT-pS473
IP: HA IB: HA
WCL IB: Flag

Fig. S5a

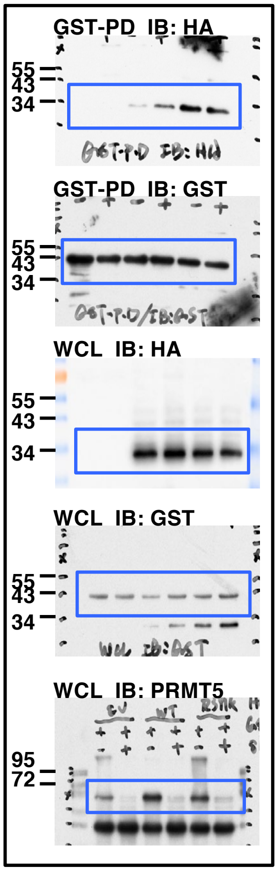

Fig. S5b

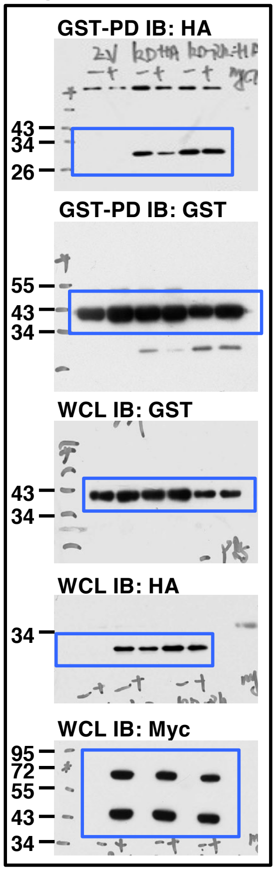

Fig. S5c

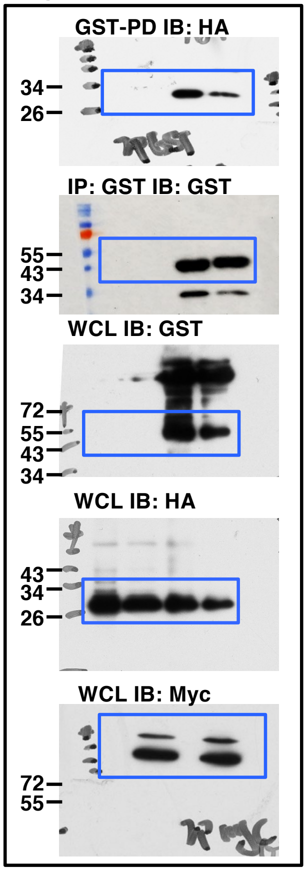

Fig. S5e

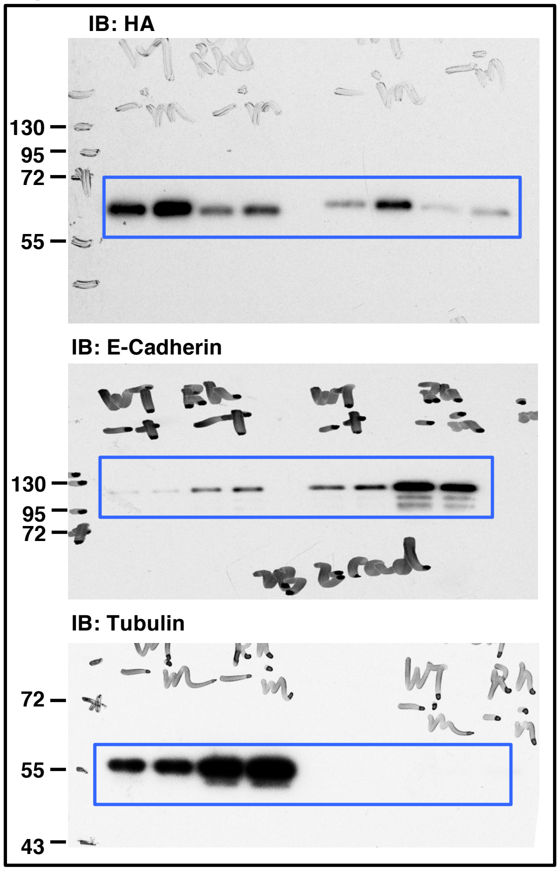

Fig. S5f

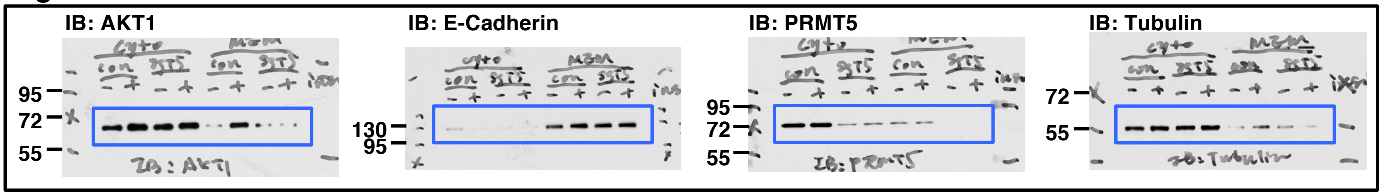

Fig. S6a

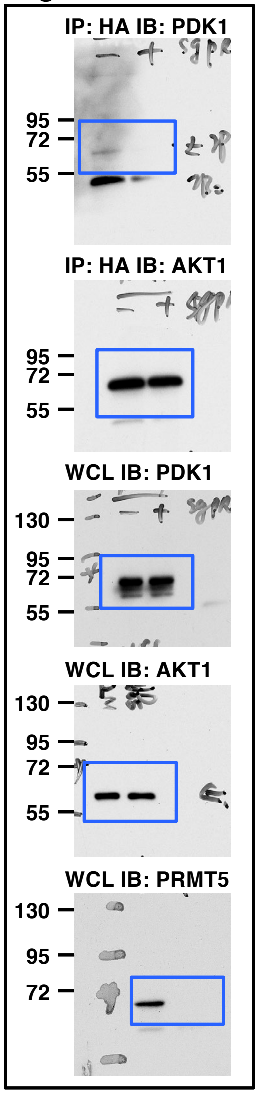

Fig. S6b

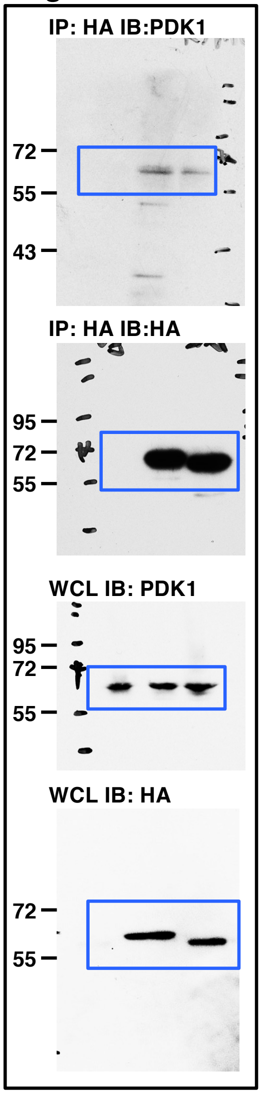

Fig. S6c

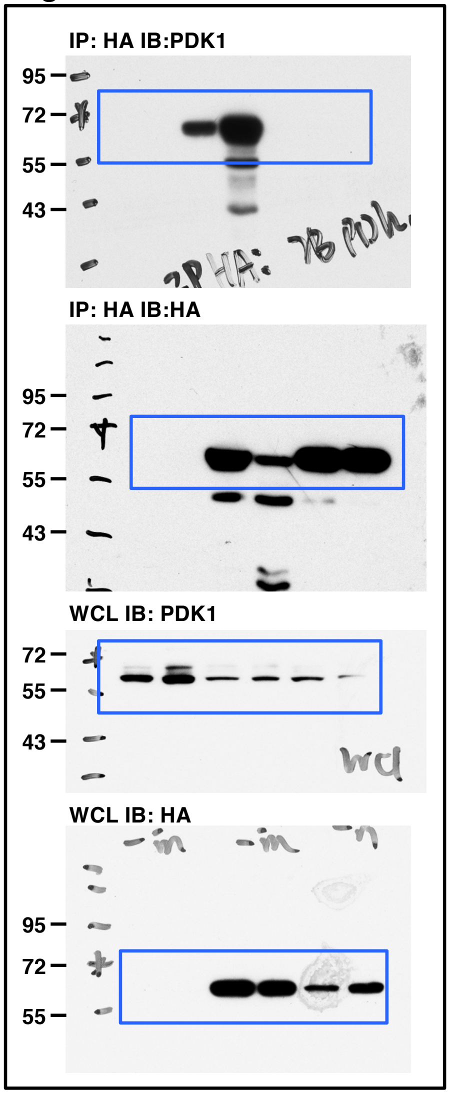

Fig. S6d

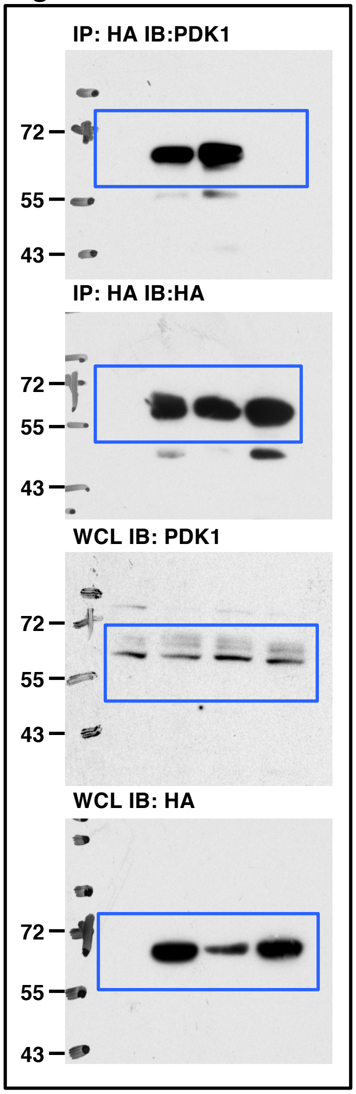

Fig. S6e

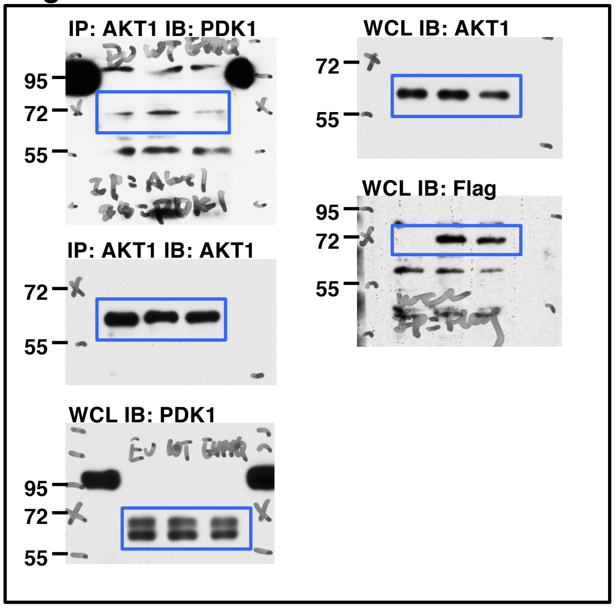

Fig. S6f

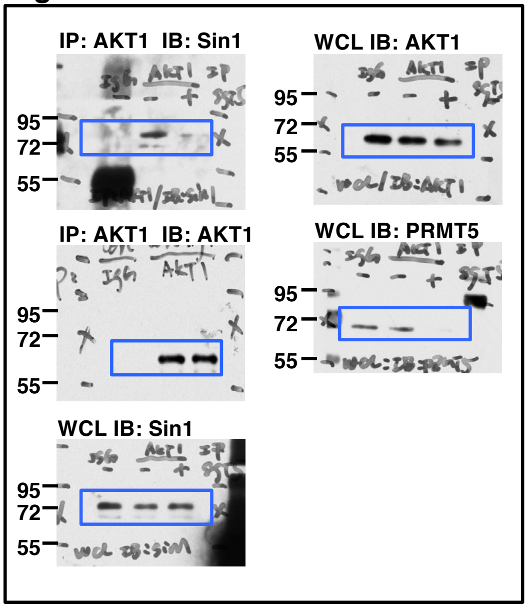

Fig. S6g

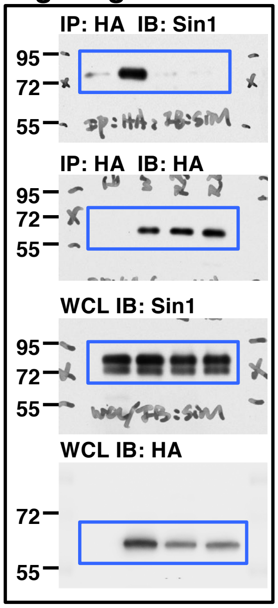

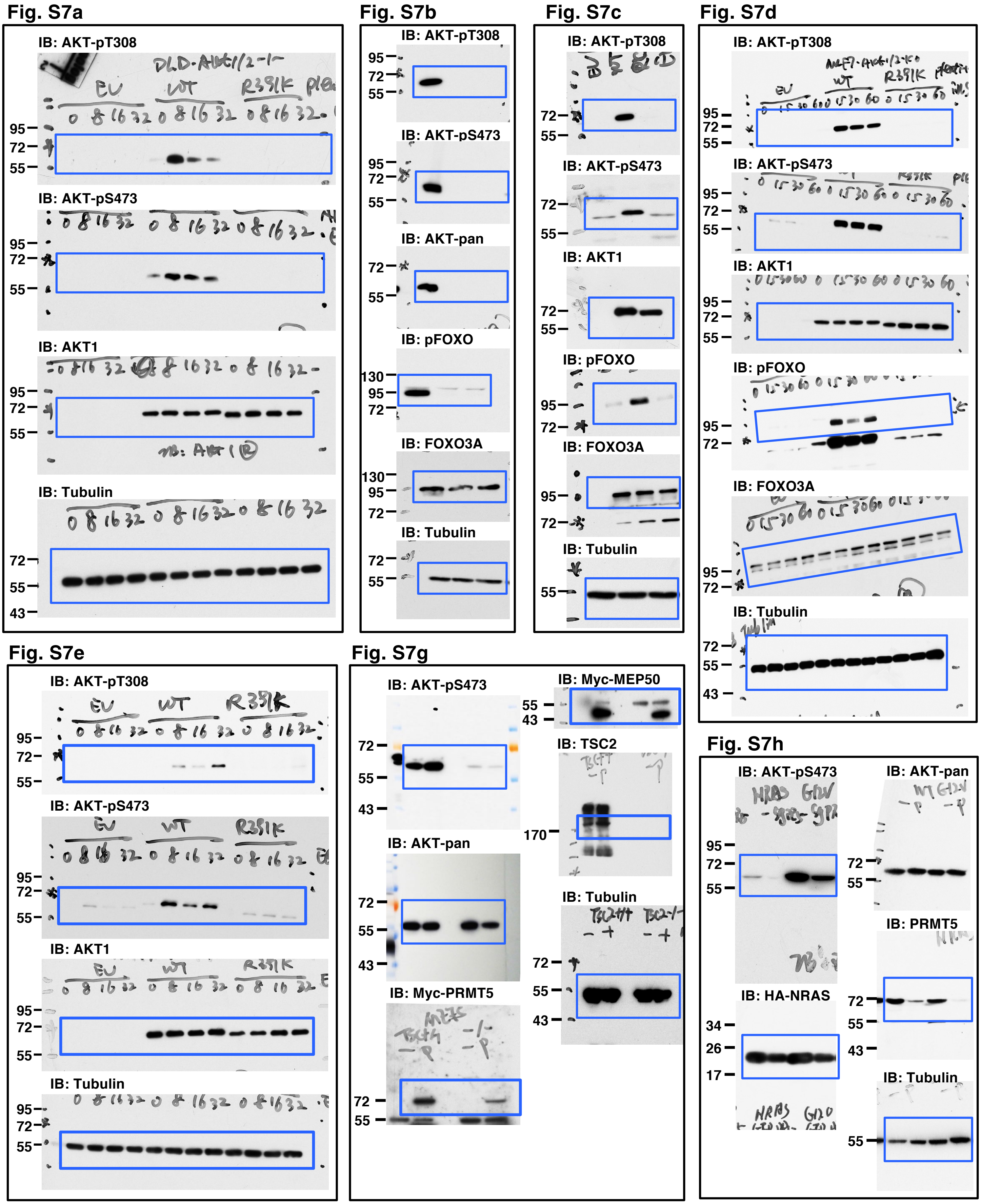

Fig. S7a
IB: AKT-pT308
IB: AKT-pS473
IB: AKT1
IB: Tubulin
Fig. S7b
IB: AKT-pT308
IB: AKT-pS473
IB: AKT-pan
IB: pFOXO
IB: FOXO3A
IB: Tubulin
Fig. S7c
IB: AKT-pT308
IB: AKT-pS473
IB: AKT1
IB: pFOXO
IB: FOXO3A
IB: Tubulin
Fig. S7d
IB: AKT-pT308
IB: AKT-pS473
IB: AKT1
IB: pFOXO
IB: FOXO3A
IB: Tubulin
Fig. S7e
IB: AKT-pT308
IB: AKT-pS473
IB: AKT1
IB: Tubulin
Fig. S7g
IB: AKT-pS473
IB: Myc-MEP50
IB: TSC2
IB: AKT-pan
IB: Tubulin
IB: Myc-PRMT5
Fig. S7h
IB: AKT-pS473
IB: AKT-pan
IB: PRMT5
IB: HA-NRAS
IB: Tubulin

Fig S8i

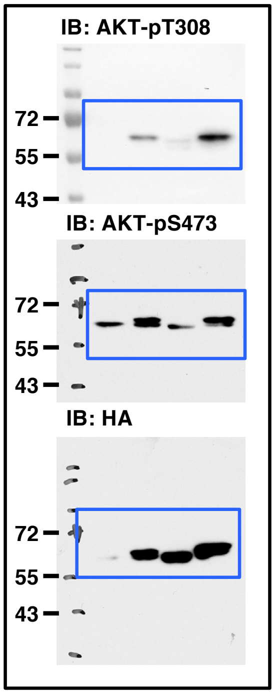

Fig. S8l

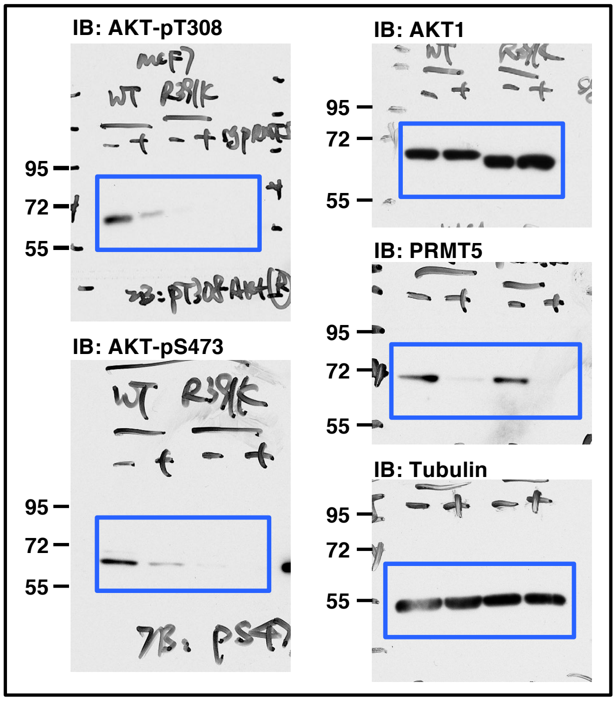

Supplement: Supplementary file 4 — Source Data [file 41467_2021_23833_MOESM4_ESM.zip › Uncropped immunoblots.pdf]
